# Metal-oxide precipitation influences microbiome structure in hyporheic zones receiving acid rock drainage

**DOI:** 10.1128/aem.01987-23

**Published:** 2024-02-23

**Authors:** Beth Hoagland, Kalen L. Rasmussen, Kamini Singha, John R. Spear, Alexis Navarre-Sitchler

**Affiliations:** 1 Department of Geology and Geological Engineering, Hydrologic Science and Engineering Program, Colorado School of Mines, Golden, Colorado, USA; 2 S.S. Papadopulos & Associates, Inc., Rockville, Maryland, USA; 3 Department of Civil and Environmental Engineering, Colorado School of Mines, Golden, Colorado, USA; University of Michigan, Ann Arbor, Michigan, USA

**Keywords:** hyporheic zone, acid rock drainage, 16S rRNA gene sequencing, Bonita Peak Mining District

## Abstract

Streams impacted by historic mining activity are characterized by acidic pH, unique microbial communities, and abundant metal-oxide precipitation, all of which can influence groundwater-surface water exchange. We investigate how metal-oxide precipitates and hyporheic mixing mediate the composition of microbial communities in two streams receiving acid-rock and mine drainage near Silverton, Colorado, USA. A large, neutral pH hyporheic zone facilitated the precipitation of metal particles/colloids in hyporheic porewaters. A small, low pH hyporheic zone, limited by the presence of a low-permeability, iron-oxyhydroxide layer known as ferricrete, led to the formation of steep geochemical gradients and high dissolved-metal concentrations. To determine how these two hyporheic systems influence microbiome composition, we installed well clusters and deployed *in situ* microcosms in each stream to sample porewaters and sediments for 16S rRNA gene sequencing. Results indicated that distinct hydrogeochemical conditions were present above and below the ferricrete in the low pH system. A positive feedback loop may be present in the low pH stream where microbially mediated precipitation of iron-oxides contributes to additional clogging of hyporheic pore spaces, separating abundant, iron-oxidizing bacteria (Gallionella spp.) above the ferricrete from rare, low-abundance bacteria below the ferricrete. Metal precipitates and colloids that formed in the neutral pH hyporheic zone were associated with a more diverse phylogenetic community of nonmotile, nutrient-cycling bacteria that may be transported through hyporheic pore spaces. In summary, biogeochemical conditions influence, and are influenced by, hyporheic mixing, which mediates the distribution of micro-organisms and, thus, the cycling of metals in streams receiving acid-rock and mine drainage.

**IMPORTANCE:**

In streams receiving acid-rock and mine drainage, the abundant precipitation of iron minerals can alter how groundwater and surface water mix along streams (in what is known as the “hyporheic zone”) and may shape the distribution of microbial communities. The findings presented here suggest that neutral pH streams with large, well-mixed hyporheic zones may harbor and transport diverse microorganisms attached to particles/colloids through hyporheic pore spaces. In acidic streams where metal oxides clog pore spaces and limit hyporheic exchange, iron-oxidizing bacteria may dominate and phylogenetic diversity becomes low. The abundance of iron-oxidizing bacteria in acid mine drainage streams has the potential to contribute to additional clogging of hyporheic pore spaces and the accumulation of toxic metals in the hyporheic zone. This research highlights the dynamic interplay between hydrology, geochemistry, and microbiology at the groundwater-surface water interface of acid mine drainage streams.

## INTRODUCTION

The hyporheic zone, or the shallow subsurface interface surrounding stream channels where the mixing of groundwater and surface water occurs, is frequently regarded as a biogeochemical reactor with important implications for the health of stream ecosystems [e.g., references (1–3)]. The hyporheic zone harbors diverse micro- and macro-species [e.g., reference (4)] that can account for up to 95% of river ecosystem respiration (5). Furthermore, the hyporheic zone mediates dynamic biogeochemical processes that vary over space and time, such as elemental cycling, pollutant removal, and processing of organic material [e.g., references (6, 7)]. The diversity and function of microbial communities in the hyporheic zone depend on controls such as seasonal changes in streamflow (8–10), grain-size distribution of bed materials and fluid residence times [e.g., references (11–13)], the chemistry of infiltrating stream water and upwelling groundwater (14, 15), and vertical mixing (16). In streams receiving acid-rock and mine discharges, the precipitation of amorphous iron oxides and hydroxides can clog hyporheic pore spaces (17, 18), which may influence hyporheic flow and lead to an overall loss of stream and subsurface water quality. In lab-controlled reactor experiments, dynamic hyporheic-mixing processes were shown to shape microbial community structure (19). To our knowledge, few if any studies have directly investigated the links between metal-oxide precipitation, groundwater-surface water interactions, and microbial community composition in mine-impacted environments, and the influence of these processes on the storage or release of metal(loid)s from the hyporheic zone.

Abiotic and biotic process rates in redox transition zones, such as those created by groundwater-surface water interactions, are hypothesized to be inherently different than rates in permanently oxic or anoxic environments (13, 20–22). For example, hyporheic zones that received a greater fraction of stream water than groundwater also received more organic carbon and dissolved oxygen, which stimulated microbial aerobic respiration (23–25). In comparison, streams with a low rate of surface-water downwelling into the hyporheic zone were characterized by limited mixing, redox stratification, and hyporheic microbial populations that varied with geochemical gradients in the hyporheic zone (23, 26), or a distinct sequence of microbial metabolism as a function of depth (27) or concentration gradient (28). Compositional shifts in microbial communities can also be driven by differences in organic carbon across the groundwater-surface water interface, where high organic carbon concentrations from surface-water downwelling have been shown to support heterotrophic microbial communities in the hyporheic zone, whereas upwelling of carbon-poor groundwater supports autotrophic microbial communities (29). Hyporheic mixing and associated fluxes in carbon and oxygen vary seasonally, which can result in a reactive hyporheic zone that harbors microbial communities that are statistically distinct from microbial communities in shallower and deeper locations in riverbeds.

Depending on subsurface redox conditions and mixing processes, microbial activity has the potential to attenuate or release toxic metals from the hyporheic zone. In the case of arsenic cycling, for example, reducing conditions in the hyporheic zone could indirectly drive the release of arsenic (primarily as As(III)) due to the reductive dissolution of poorly crystalline Fe(III) or Mn(IV) hydroxides by dissimilatory reducing bacteria (30, 31). Under oxidizing conditions, such as when stream-water contributions to the hyporheic zone are high, attenuation of arsenic can occur due to the oxidation of Fe(II) or Mn(II) and precipitation of Fe(III) or Mn(IV) oxides in the presence of *Acidithiobacillus ferrooxidans*, *Leptospirillim ferroxidans*, and *Leptothrix* bacteria, which facilitate the adsorption or co-precipitation of arsenic (primarily as As(V), depending on the pH of interacting waters). The link between metal concentrations in streambed sediments and microbial community composition also varies seasonally, where the strongest correlation between trace-metal contamination and the abundance of metal-tolerant or metal-detoxifying phylogenies has been shown to occur during seasons when streams receive the most organic matter deposition (8). Thus, hyporheic exchange flows have important implications for how microorganisms respond to metal loads, as the mixing of these upwelling groundwater and infiltrating stream water impacts the supply of terminal electron acceptors and donors.

Previous work has highlighted that sediment surfaces versus pore space in aquifers can harbor different microbial communities with distinct physiological capabilities (32, 33). For example, *Geobacter*, a genus of the phylum Desulfobacterota and class Desulfuromonadia that respires insoluble iron-oxide substrates, were more abundantly attached to sediments than suspended in porewaters, regardless of changes to groundwater chemistry (34). Microbial colonization of *in situ* sediments versus interstitial porewaters has also been investigated in the hyporheic zone, where higher abundances of certain taxa such as *Chloracidobacteria* were found attached to surfaces rather than suspended in pore spaces and these taxa played a role in biofilm stability and function (29). Suspended- versus sediment-microbial communities can serve different ecosystem functions as well. Suspended microorganisms may help with cell dispersal among attached microbial communities (29), while the formation of microbial biofilms can be an important protection against metal loading (35). Furthermore, high solute residence times in the hyporheic zone lead to enhanced immobilization of solutes by biofilms (36). We would, therefore, expect that the partitioning of certain taxa between hyporheic waters and hyporheic sediments may influence metal and nutrient cycling in mine-impacted streams.

In this study, we investigated how hyporheic mixing processes may influence microbial community composition in two streams—Mineral and Cement Creeks—impacted by acid rock drainage and historic mining activities in the headwaters of the Animas River watershed near Silverton, Colorado, in the Bonita Peak Mining District Superfund Site. In the previous work, the hyporheic zones at Mineral Creek and Cement Creek were used to build a conceptual model for “well-connected” and “poorly connected” stream-groundwater systems, respectively (37). These two models were representative of the distinct differences that these hyporheic systems had on metal(loid) concentration and export. In the well-connected system of Mineral Creek, hyporheic mixing facilitated dissolved-oxygen penetration deep into the subsurface that triggered the precipitation of metal particles or colloids and inhibited the entry of dissolved metals to the stream. In the poorly connected Cement Creek system, hyporheic sediments were an important regulator of metal(loid) concentrations in the shallow subsurface, but the hyporheic zone was not as important for overall stream metal(loid) export (37).

Given the previously documented role of these two hyporheic systems on metal(loid) export, the purpose of this study was to (a) determine the distribution and potential function of microorganisms in these two systems and (b) investigate the link between groundwater-stream connectivity and microbially mediated cycling of metals in mine-impacted hyporheic zones. Given the oxic, well-mixed, and neutral pH hyporheic zone at Mineral Creek, we hypothesized that the microbial diversity would be greater compared to Cement Creek and include aerobic heterotrophs that contribute to the natural attenuation of toxic metals. In comparison, we hypothesized that the limited groundwater-stream water connectivity, acidic pH, and metal-rich conditions at Cement Creek would limit microbial diversity and influence the distribution of microbial communities in the hyporheic zone.

## MATERIALS AND METHODS

### Site background and hyporheic zone characteristics

Mineralized veins containing iron-rich sulfide minerals are prevalent throughout the Animas River watershed, which supported a widespread gold and silver mining industry in this region beginning in the late 1800s and lasting for over a century (38). While there is no longer active mining within the area, the three drainages that comprise the Animas River headwaters—Mineral Creek, Cement Creek, and the Upper Animas River—receive diffuse, metal-rich drainages of 20.4 million liters per day (39). Furthermore, these streams are currently the site of an U.S. Environmental Protection Agency (EPA) Superfund Site known as the Bonita Peak Mining District, where the focus is on stabilizing and reducing loads of metals such as Al, As, Cd, and Zn from 48 historic mines or mining-related features (40). The Superfund Site was established soon after the Gold King Mine spill in August 2015 when an accidental breach of a tunnel connected to the legacy Gold King Mine led to the release of a slug of approximately 11 million liters of acidic mine drainage into the Animas headwaters.

**Fig 1:**
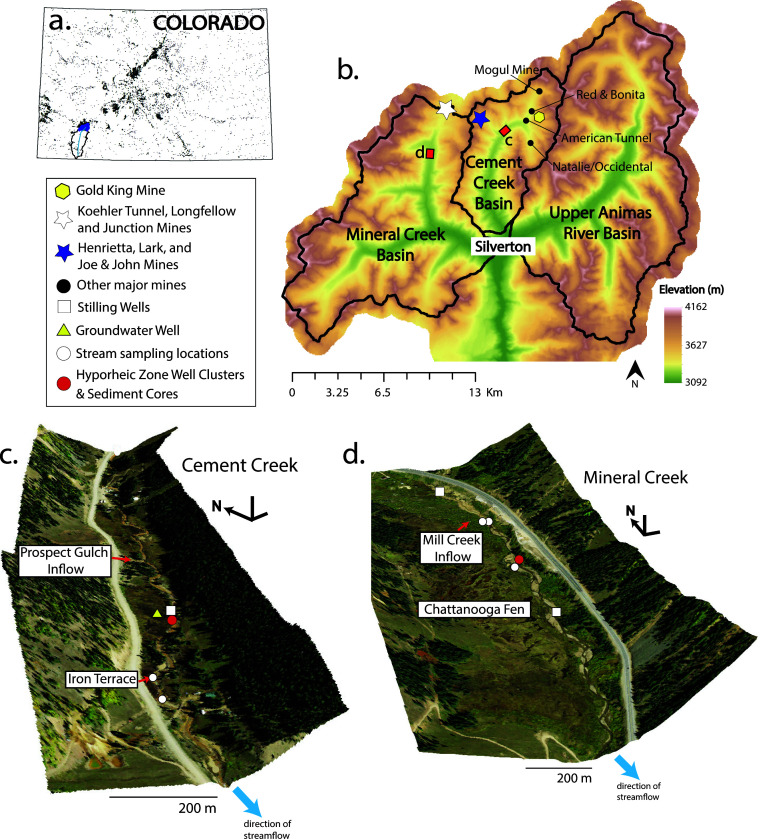
Locations of the Mineral Creek and Cement Creek basins in (**a**) the headwaters of the Animas River watershed in southwest Colorado (as denoted by the dark blue highlighted region). Black points in (**a**) represent mine-related features in the state of Colorado (43). Detailed views of the study reach as outlined by red squares in (**b**) are presented at a 65° viewing angle for (**c**) Cement Creek at Prospect Gulch and (**d**) Mineral Creek at Chattanooga Fen. The Shuttle Radar Topography Mission (SRTM) 30 m digital elevation model was used to map the elevation above mean sea level in (**b**). Aerial imagery for (**c**) and (**d**) is from the USDA National Agriculture Imagery Program (NAIP) dated 20 September 2017.

From May to October 2019, we investigated microbial communities in the hyporheic zones of Cement and Mineral Creeks. Sediment, stream, and hyporheic porewater samples were collected for 16S rRNA gene sequencing (as described in the “Hyporheic zone well clusters and sediment sampling,” “Deployment of *in situ* microcosms,” and “Sampling for geochemical and microbial analyses” sections) from two stream reaches with distinct hyporheic and geochemical characteristics—Cement Creek downstream of Prospect Gulch and Mineral Creek near Chattanooga Fen (Fig. 1). Throughout the remainder of the text, these sites will be referred to simply as Cement Creek and Mineral Creek. Cement Creek is characterized by pH ranging from approximately pH 3 to 4.5 and elevated dissolved concentrations of metals such as Fe, Mn, and Al, whereas Mineral Creek has near-neutral pH and dissolved concentrations of trace metals that are several orders of magnitude lower than Cement Creek [e.g., references (37, 41, 42)]. In 2019, stream flow (*Q*) at the stream outlets ranged from winter baseflow of *Q* ∼ 0.3 m^3^ s^−1^ to spring snowmelt as high as *Q* ~ 15 m^3^ s^−1^ in Cement Creek and winter baseflow of *Q* ∼ 0.4 m^3^ s^−1^ to spring snowmelt as high as *Q* ~ 34 m^3^ s^−1^ in Mineral Creek (41).

Both streams are located near or downstream of mine drainages. Mineral Creek is located downstream of the Koehler Tunnel and Junction and Longfellow Mines, which are priority cleanup sites of the Bonita Peak Mining District, and adjacent to a rare iron fen [iron fens represent <1% of all fens in the Rocky Mountains (44)]. This iron fen, known as the Chattanooga Fen complex, has metal concentrations as high as ~20 mM Al, ~7.8 mM Fe, and ~30 mM Mn (37). The Cement Creek site is also adjacent to an iron fen that is smaller, less vegetated, and ephemerally saturated compared to the Chattanooga Fen complex at Mineral Creek. The Cement Creek site is located downstream of some of the largest draining mines in the Animas River headwaters, including the Mogul Mine, Red and Bonita Mine, Gold King Mine, and American Tunnel, as well as the Henrietta, Lark, and Joe and John Mines located in Prospect Gulch, some of which are priority remediation sites of the U.S. EPA and Colorado Department of Public Health and Environment (CDPHE).

The streambed properties differ at Cement and Mineral Creeks and impact the hyporheic exchange processes at these two sites. The streambed of Cement Creek contains large cobbles and boulders cemented together by orange iron oxide and ferricrete precipitates. The ferricrete, or clastic sedimentary conglomerates cemented together by crystalline and amorphous iron oxyhydroxides, is composed of minerals such as schwertmannite, goethite, and jarosite and forms as a result of acidic, reducing groundwaters interacting with oxygenated surface waters or the atmosphere (45, 46). The extensive ferricrete precipitation clogs the streambed and banks at Cement Creek, which was found to create low streambed hydraulic conductivity and average linear velocities of infiltrating stream water and limit the hyporheic storage area to ~0.1 m^2^ at low flow and ~0.4 m^2^ at high flow (47). In comparison, the high hydraulic conductivity and average linear velocities of the large-cobbled streambed at Mineral Creek facilitated hyporheic mixing and a large hyporheic storage area (~0.6 m^2^ at low flow and ~1.8 m^2^ at high flow) [Table 1; (37)].

**TABLE 1:** Summary of geochemistry and hyporheic characteristics at Cement and Mineral Creeks

	Mineral Creek		Cement Creek	
	High flow^*a*^	Low flow	High flow	Low flow
Depth-averaged pH	6.8^*a*^	6.4	4.0^*a*^	3.9
Depth-averaged dissolved oxygen (DO)	7.8^*a*^	9.1	4.5^*a*^	4.1
Depth-averaged sediment pH^*b*^	5.6 ± 0.3^*c*^	–	3.8 ± 0.1	–
Hyporheic storage area (*A*_s_, m^2^)^*d*^	1.8	0.6	0.4	0.1
Hyporheic exchange rate (*α*, s^−1^)^*d*^	0.005	0.004	0.001	0.001
Residence time in storage zone, *T*_sto_ (h)^*d*^	2.7	2.8	4.1	2.7

^*a*^ Includes measurements from two sampling campaigns, one from 2019 and 2020.

^*b*^ Average sediment pH calculated for the same samples used for the construction of the *in situ* microbial samplers, including the 0–10 and 10–20 cm depth intervals for Cement Creek and the 10–20, 30–40, and 60–67 cm intervals for Mineral Creek.

^*c*^ Standard deviation calculated for duplicate measurements for all samples at Mineral Creek (*n* = 12) and Cement Creek (*n* = 8).

^*d*^ Hyporheic zone parameters from (47) for Cement Creek and (37) for Mineral Creek.

### Hyporheic zone well clusters and sediment sampling

A cluster of three monitoring wells was installed in the streambed of each stream reach to evaluate microbial community composition as a function of depth in sediments and porewaters of the hyporheic zone. In addition to microbial sampling, the monitoring wells were used to measure vertical hydraulic gradients, perform slug tests, and sample porewater chemistry in previous work (37, 47). Hyporheic zone monitoring wells extended to depths of 28, 44, and 58 cm at Cement Creek (37.88049°, −107.66814°) and to depths of 20, 40, and 68 cm at Mineral Creek (37.86970°, −107.72387°) (Fig. 1c and d). The wells were constructed from PVC with a 19 mm inner diameter and a 0.2 mm slotted screen comprising the bottom 10 cm of each well and were installed on 18 July 2019 in Cement Creek and 21 May 2019 in Mineral Creek. A high-flow event washed out the initial installation of well clusters at Cement Creek, which delayed subsequent sediment sampling.

The well clusters were left undisturbed for longer than 2 weeks prior to sediment sampling. Sediment samples were collected from exposed parts of the streambed of Cement Creek on 31 July 2019 and Mineral Creek on 21 June 2019 (referred to from here on as the “streambed sediments”). Although samples were collected ~1 month apart at the two sites, the differences in streamflow and temperature between the sampling dates in June and July were relatively small compared to seasonal differences in temperature and streamflow between winter and summer in this snowmelt-dominated terrain. Sediment samples were collected using a soil auger with depth in 10 cm increments. In Cement Creek, streambed sediment samples were collected from two locations approximately 2 and 5 m away from the monitoring well cluster in a dry portion of the stream channel. Given ferricrete precipitation along the banks of Cement Creek, we were unable to collect samples below a depth of 20 cm or immediately adjacent to the well installation site. In Mineral Creek, sediment samples were collected to a depth of 68 cm and sediments from each depth interval were homogenized and split into subsamples.

One set of subsamples (~30 g each) was placed in sterile plastic bags for analysis of sediment pH and for archival with the System for Earth Sample Registration (SESAR, IGSNs included in Table S1). The sediment pH was determined by preparing a sediment slurry in the lab of 10 g sediment mixed with 20 mL of deionized water and measuring the pH of the slurry with a pH meter (48). The second set of subsamples (~10 g) was placed in sterile centrifuge tubes for DNA extraction and subsequent 16S rRNA gene sequencing of the microbial community composition in the sediments prior to deployment in the well clusters (i.e., *t* = 0 d). Both subsamples were immediately placed on ice and frozen at −20°C within 3 h of collection.

### Deployment of *in situ* microcosms

A third subset of the streambed sediment samples was used to make *in situ* microcosms for microbial community characterization (Table 2). The *in situ* microcosms were designed to sample microbial community composition and geochemistry after incubation in the hyporheic zones of the two hydro-geochemically distinct streams. It was beyond the scope of this study to use the microcosms to measure functional changes or temporal shifts in geochemical conditions. Approximately 2 g of the streambed sediment was placed in a nylon mesh bag (<100 µm opening) using sterilized tools. Bags were then deployed in duplicate or triplicate within each hyporheic zone monitoring well at each site. For Mineral Creek, the streambed sediment sample used in the *in situ* microcosm corresponded to the depth interval of the screened portion of the well it was being deployed in. For example, the 11–20 cm below ground surface (bgs) streambed sediment was deployed in the shallowest well where the screened portion of the well spanned from 10 to 20 cm depth (Table 2). Given that we were unable to auger below a depth of 20 cm in Cement Creek, the 0–10 cm streambed sediment interval was deployed in the shallow (18–28 cm bgs) and intermediate wells (34–44 cm bgs), while the 10–20 cm interval was deployed in the deepest well (48–58 cm bgs). The *in situ* microcosms were deployed in the Cement Creek wells for 50 days (removed on 21 September 2019) and deployed in the Mineral Creek wells for 98 days (removed on 27 September 2019). Upon removal, samples were immediately placed in sterilized centrifuge tubes, put on ice, and frozen at −20°C within 3 h of collection. The sediments deployed in the well clusters are referred to from here on as the “microcosm sediments.”

**TABLE 2:** Sample collection depths for the streambed sediment cores and corresponding depths of deployment in the well clusters for the *in situ* microcosm sediments

Stream system	Streambed sample depth interval (cm)	Depth interval for *in situ* microcosm sediment (cm)	Microcosm depth classification
Mineral Creek	11–20	10–20	Shallow
	30–40	30–40	Intermediate
	60–68	60–68	Deep
Cement Creek	0–10	18–28	Above Ferricrete
	0–10	34–44	Near Ferricrete
	10–20	48–58	Below Ferricrete

### Sampling for geochemical and microbial analyses

Descriptions of the methods and results for the stream and hyporheic porewater chemistry were previously described in reference (37). In summary, geochemical characterization of both the stream and wells was based on field measurements of temperature, pH, dissolved oxygen, conductivity, ferrous iron (Fe^2+^), and alkalinity, as well as analysis of samples for total metals (unfiltered), dissolved metals (filtered <0.2 µm, Nylon), and anions (filtered <0.2 µm, Nylon). All water samples were collected according to standard methods for groundwater (49) and stream water (50).

For this study, additional samples were collected for analyses of total organic carbon (TOC) and microbial community composition. Samples for TOC and microbial community composition were collected on the same dates as the *in situ* microcosm deployment and removal. Water samples for TOC were unfiltered, stored in combusted (400℃) amber glass vials, preserved using hydrochloric acid (10%, vol/vol), and refrigerated at 4℃ until analysis within 1 month of sample collection. TOC samples were analyzed by a Shimadzu TC-Analyzer at the Colorado School of Mines Advanced Water Technology Center (AQWATEC). Sediment samples for DNA extraction and sequencing were collected from the streambeds at both sites (as described in the “Hyporheic zone well clusters and sediment sampling” section), as well as sediments from an iron terrace at Cement Creek only (Fig. 1c). Stream samples (*n* = 2 at each site) were collected as grab samples. Hyporheic porewaters (*n* = 6, three depths at each site on two sampling dates) and Cement Creek groundwater (*n* = 2) were sampled from the hyporheic well clusters and a shallow groundwater well, respectively, at a low pumping rate (<200 mL/min) with a peristaltic pump. The Cement Creek groundwater samples were collected from an existing well within a small iron fen adjacent to the well cluster location. Although water samples collected from the streambed could be representative of upwelling groundwater, all water samples collected from the hyporheic well clusters are referred to throughout the text as “porewater” samples. The stream, porewater, and groundwater samples were collected for DNA extractions via field filtration of 1 L of water through 0.2 µm filters (Supor-200 polyethersulfone). Filters were immediately placed on ice and frozen until analysis. The water samples were also analyzed using inductively coupled plasma-mass spectrometry and ion chromatography for geochemical analytes, as described in reference (37).

### 16S rRNA gene analyses

Total community DNA was extracted from streambed and microcosm sediments and filters using the ZymoBIOMICS DNA Miniprep Kit (Cat No. D4300) and the binding preparation protocol for soil samples described in the kit. Extraction yields for each sample are reported in Table S2. A quantifiable amount of DNA was extracted from every sample. DNA extraction yields were lower in Cement Creek compared to Mineral Creek, as is common for samples containing high concentrations of iron or abundant iron-rich clays (51). Extraction and sequencing methods were completed following the protocol in references (52, 53). Assurance of quality control was ensured by performing DNA extractions on field replicates (Table S3) and three controls (i.e., nuclease-free water). The final pooled library was submitted for high-throughput sequencing on the Illumina MiSeq platform using the PE250 V2 chemistry method (Illumina, San Diego, CA, USA) at the Duke Center for Genomic and Computational Biology. See the Supplementary Information for additional detail.

Sequenced reads were processed as described previously in reference (54). In brief, sequence reads were demultiplexed using adapterremoval2 (55). The reads were then checked for chimeras and separated into single DNA sequences, or amplicon sequence variants (ASV), using the DADA2 algorithm (56). Sequences were aligned and a phylogenetic tree was constructed using the QIIME2 pipeline (57). Taxonomy was assigned using the DADA2 release of the SILVA 16S rRNA gene database [v. 132 (58)].

Statistical methods were used to evaluate differences in the microbial community composition of the hyporheic zone at Cement Creek and Mineral Creek. Alpha diversity was evaluated using the Shannon-Wiener Diversity Index, which was calculated using the R package phyloseq (59) and data visualization package ggplot2 (60). Microbial abundance heatmaps were created using ampvis2 (61). Beta diversity (community variance between samples) was calculated in phyloseq on rarefied data sets using weighted unique fraction metric (unifrac) distances, which calculates the dissimilarity between taxa based on phylogenetic distances and abundance information (62). The weighted unifrac distance was then related to environmental variables using the vegan program in R (63). Constrained canonical analysis of principal coordinates (CAP) was used to test the relationship between microbial community composition and environmental data.

Due to the range of sequencing depth across samples (Fig. S1), we rarefied (i.e., normalized sequencing depth) samples for several different subsets of the sample data using ggrare in the phyloseq package (64). We rarefied to even sampling depths for a subset of data that included all samples (i.e., from both sites and including groundwater, hyporheic porewater, and sediment samples), for subsets by sampling location (i.e., Mineral Creek and Cement Creek), and for subsets that included only the porewater samples or only the sediment samples. Rarefaction did not appear to have an impact on the relative differences in observed alpha diversity (Fig. S2). Pairwise permutational analysis of variance (PERMANOVA) was used to test whether the beta diversity calculations were statistically significant (*P*-value < 0.05) as a function of sample type (porewater versus microcosm sediment) in each stream with the *adonis* function in vegan (63). Only two samples were collected at each depth, except for the depths where duplicates were collected, and thus, there were not enough samples to conduct a reliable PERMANOVA analysis and test the significance of sample clusters as a function of depth. All sequences were submitted to the National Center for Biotechnology Information (NCBI) Sequence Read Archive (SRA) database under BioProject number PRJNA952531.

### Differential abundance analysis

Differential abundance analysis was performed on 16S rRNA gene data to test the null hypothesis that the mean abundance of taxa in the *in situ* microcosm sediment samples was equally abundant compared to the mean abundance of taxa present in the porewater samples. By testing this hypothesis, the goal was to evaluate the differential abundance of attached and suspended microbial communities in the hyporheic zones. In our analysis, microcosm sediment species abundances were the reference condition and porewater species abundances were the comparison condition. Significance was based upon a log_2_ fold change [log_2_FC = log_2_(mean abundance (microcosm sediments)/mean abundance(porewaters))] and a corrected *P*-value (see the Supplementary Information for more detail). Differential abundances characterized by a log fold change (lfc) ≥2 and a corrected *P*-value ≤ 0.01 were considered significantly different, and the null hypothesis that microcosm sediments and porewaters have the same species abundance could be rejected. The analysis was conducted in R using the DESeq2 package (65). Three samples were identified as outliers using principal component analysis and Cook’s cutoff distances and removed from the differential abundance analysis (Table S4). After outlier identification and removal, seven porewater and five microcosm sediment samples remained for Mineral Creek and five porewater and four microcosm sediment samples remained for Cement Creek. Pre-filtering was conducted to remove taxa with low read counts, where only taxa with read counts greater than or equal to three in more than three samples were kept in the analysis.

## RESULTS

### Geochemical conditions in Cement and Mineral Creeks

**Fig 2:**
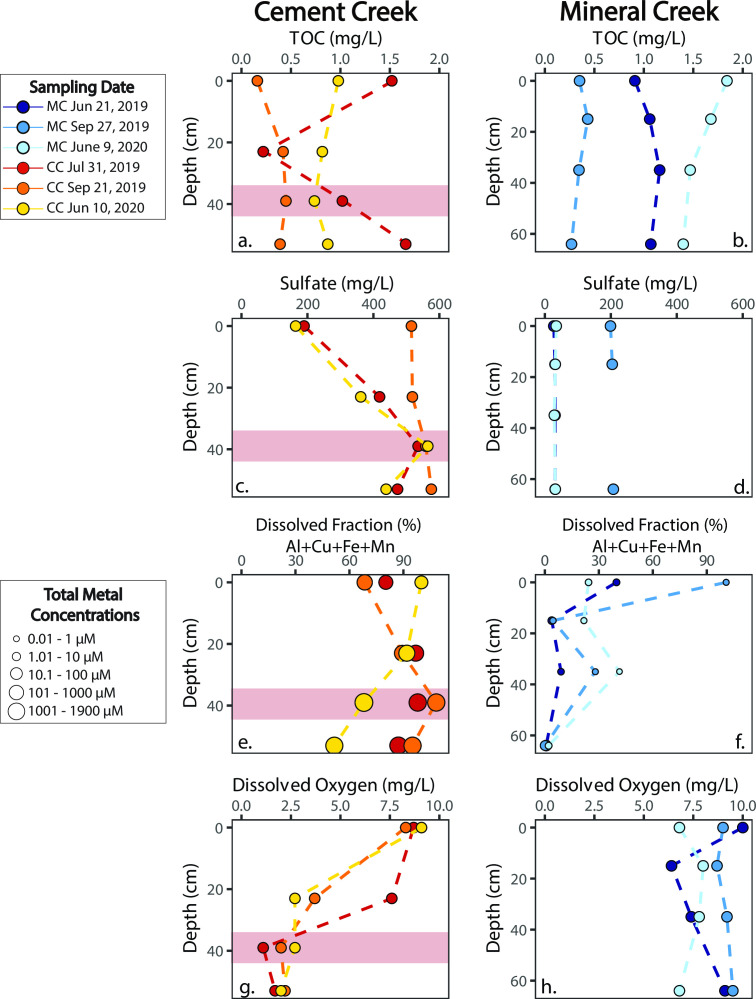
Depth profiles for the Mineral Creek and Cement Creek well clusters for (**a and b**) total organic carbon, (**c and d**) sulfate, (**e and f**) fraction of dissolved to total metals calculated based on the sum of Al, Cu, Fe(III), and Mn concentrations, and (**g and h**) dissolved oxygen. The size of the points in (**c**) scale with total metal concentrations as defined in the explanation. Red-shaded region highlights the approximate hypothesized location of the ferricrete layer.

Based on previous work at Cement Creek, a low-permeability layer of ferricrete was identified at ~45 cm below the streambed that separated a small hyporheic zone above the ferricrete from shallow groundwater beneath the ferricrete (47). Geochemistry differs above and below the ferricrete layer, where dissolved oxygen concentrations decrease and dissolved metal concentrations such as Al, Fe(III), and SO_4_^2−^ increase with depth (Fig. 2a, c and g). Metals are present primarily as the dissolved phase (<0.2 µm in size) in the Cement Creek hyporheic zone, where the fraction of dissolved to total metals is >50% across all depths (Fig. 2e).

For Mineral Creek, water chemistry in the hyporheic zone is relatively homogenous (37). Dissolved oxygen concentrations are consistently high and sulfate concentrations are invariable across all sampling depths (Fig. 2d and h); however, the ratio of dissolved to total metals decreases with depth (Fig. 2f). The high fraction of particulate metals in the wells of Mineral Creek (<50% across all hyporheic samples and depths; Fig. 2f) was previously attributed to the mixing of metal-rich groundwater and oxygen-rich stream water in a large hyporheic zone [Table 1; (37)]. This mixing at Mineral Creek likely caused the precipitation of metal-oxide particulates and/or colloids (>0.2 µm in size) in hyporheic pore spaces.

### Microbial diversity in hyporheic sediments and porewaters

**Fig 3:**
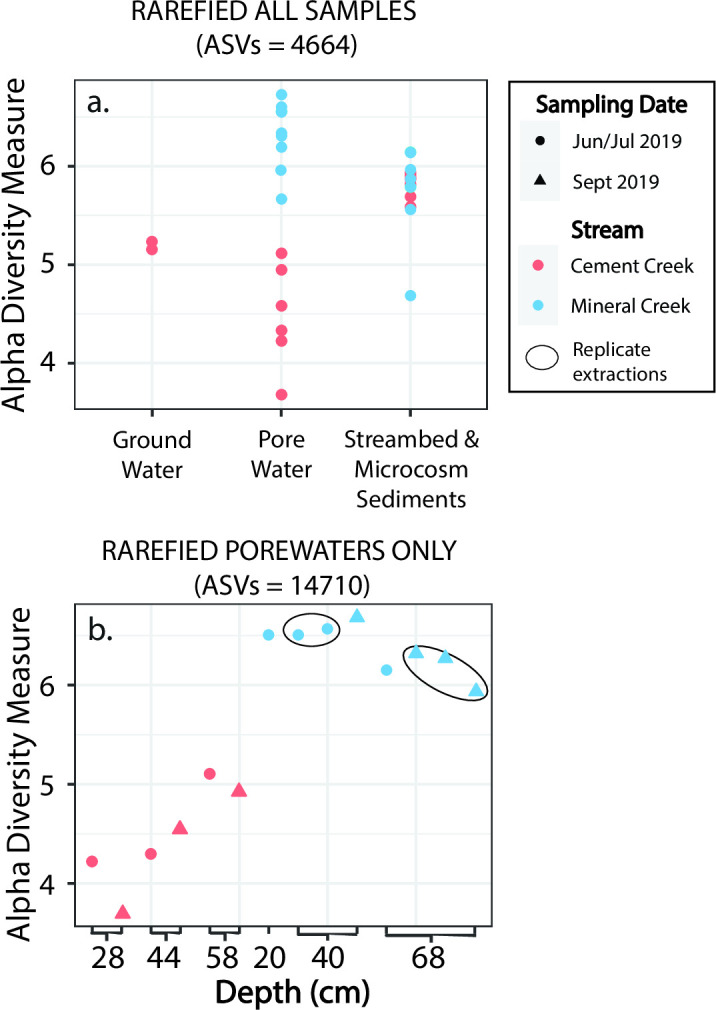
Shannon diversity index based on 16S rRNA gene sequencing data from hyporheic zone well cluster. (**a**) Shannon diversity calculated on all samples rarefied to the lowest amplicon sequence variant (ASV) of all groundwater, porewater, and streambed and microcosm sediment samples from both Mineral and Cement Creeks (i.e., 4,664 counts, Fig. S3). (**b**) Shannon diversity calculated for rarefied porewater samples only for both Mineral and Cement Creeks (i.e., 14,710 counts, Fig. S1). Technical extraction replicates (black ovals) exhibit good agreement in alpha diversity. Circles in (**b**) represent samples collected in June and July 2019 during initial installation of the well clusters and triangles represent porewaters collected during *in situ* microcosm sediments in September 2019.

Alpha diversity (within sample diversity) based on the Shannon-Wiener Index indicates that taxonomic diversity differed by both stream site (Cement and Mineral Creek) and sample type (sediments and hyporheic porewaters). This was further supported by PERMANOVA calculations, which indicate that the variance between Cement Creek and Mineral Creek samples and the variances between sediment (streambed and microcosm sediments) and porewater samples were statistically significant (Table 3). Cement Creek had lower alpha diversity in the porewater samples compared to Mineral Creek, whereas alpha diversity in the sediment samples (streambed and microcosm) at the two sites was similar (Fig. 3).

**TABLE 3:** PERMANOVA results for comparisons between each sample type and the stream system or depth^*a*^

Analysis	Rarefaction subset	Comparison	*F-*stat	*R* ^2^	*P*-value
Weighted UniFrac	All samples	Stream	6.510	0.246	0.001
		Sample type	3.982	0.166	0.002
	Cement Creek	Sample type	8.29	0.675	0.003
	Mineral Creek		6.115	0.357	0.003
Unweighted UniFrac	All samples	Stream	4.888	0.196	0.001
		Sample type	2.445	0.109	0.005
	Cement Creek	Sample type	1.953	0.151	0.001
	Mineral Creek		5.143	0.255	0.001

^*a*^ Calculations completed in R with the adonis function in the vegan package and with outlying samples removed.

#### Cement Creek

**Fig 4:**
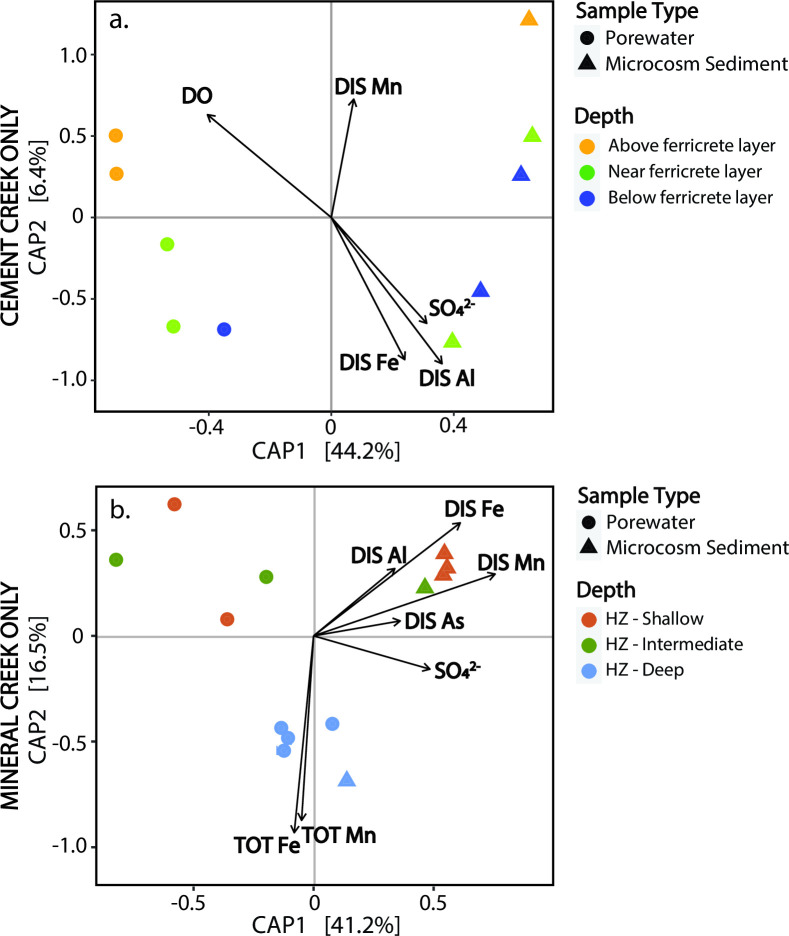
Statistical analysis of beta diversity using CAP ordination and weighted Unifrac distancing for microbial communities in (**a**) Cement Creek and (**b**) Mineral Creek for porewater and microcosm sediment samples. Plots estimated using unweighted Unifrac distancing are included in Fig. S4. Geochemical vectors represent parameters that influence microbial community composition. The length of vectors represents the magnitude of influence on plotted points and the proximity of points to the vectors represents correlation. DO, dissolved oxygen; TOT, total concentration; DIS, dissolved concentration; HZ, hyporheic zone.

The alpha diversity in porewater samples increased with depth (Fig. 3b) and the deepest porewater samples had alpha diversity similar to the shallow groundwater samples collected in an adjacent iron fen at Cement Creek. Changes in microbial community composition of the porewaters with depth were also apparent in the beta diversity (across sample group diversity), where the distribution of porewater samples along the secondary axis (CAP2) was large and related to the sampling depth (Fig. 4a).

The beta diversity of the microcosm sediment samples differed from that of the porewater samples (see clustering along CAP1 in Fig. 4a). The beta diversity of the initially collected streambed sediments and microcosm sediments differed for some samples as well, indicating that the incubation period of 50–100 days was sufficient time for shifts in the microbial community from the original sediment to occur (Fig. S3). In these dynamic hyporheic systems, it is possible that microbial community composition may continue to change throughout the year because of seasonal fluctuations in water levels, temperature, and metal loadings; however, evaluation of the impacts of seasonality on microbial communities was beyond the scope of the present study.

Microbial porewater and microcosm sediment samples collected from above the ferricrete layer at Cement Creek cluster in the positive CAP2 space and correspond with the dissolved oxygen (DO) and dissolved Mn environmental vectors, whereas the microbial samples collected below the ferricrete layer were correlated with dissolved concentrations of Al, Fe(III), and SO_4_^2−^ (Fig. 4a). These same environmental variables created strong chemical gradients below the Cement Creek streambed (37).

#### Mineral Creek

Similar to Cement Creek, the beta diversity of Mineral Creek samples clustered based on sample type (Fig. 4b). The distinct beta diversity clusters for porewaters and microcosm sediments were supported by PERMANOVA calculations, which indicate that the variance between microcosm sediment and porewater samples (*P* ~ 0.003) was statistically significant (based on a significance threshold of *P* ≤ 0.05) and not due to random chance (Table 3).

Three distinct beta diversity clusters were apparent for Mineral Creek samples: shallow and intermediate porewater samples, shallow and intermediate microcosm sediment samples, and deep porewater and microcosm sediment samples (Fig. 4b). Beta diversity of the deep microcosm sediment and porewater samples corresponded with environmental vectors for total (i.e., unfiltered) Mn and Fe concentrations (Fig. 4b). The microcosm sediment and porewater samples collected from the shallowest well were more positive along the CAP2 axis and corresponded with environmental vectors for dissolved Fe, Mn, Al, and As concentrations (Fig. 4B;) (37).

### Hyporheic microbial community composition

#### Microbial community composition with depth: Cement Creek

**Fig 5:**
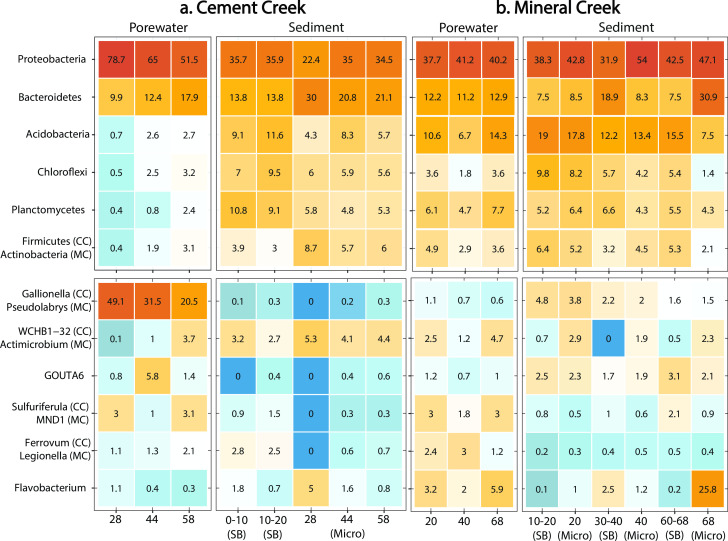
Percent relative abundance of the top six bacteria/archaea phyla (top row of panels) and genus (bottom row of panels) parsed by sample type for (**a**) Cement Creek and (**b**) Mineral Creek. Blue shades represent low abundance reads and red shades represent high abundance reads with values representing the percent read abundance. Heat maps for negative and positive controls are included in Fig. S7, and heat maps for stream water samples are in Fig. S8 and S9. The 0–10 and 10–20 cm depths in Cement Creek and the 10–20, 30–40, and 60–68 cm depths in Mineral Creek represent the samples collected from the streambeds (SB) and used for the microcosm (Micro) sampling bags in the hyporheic wells. Phyla and genus with “CC” or “MC” in parentheses represent the phyla or genus for Cement Creek and Mineral Creek, respectively, in that row. If a “CC” or “MC” label is not included, this indicates the genus or phyla in that row are the same for Cement Creek and Mineral Creek.

Cement Creek porewaters are associated with a decrease in the relative abundance of microorganisms belonging to the Proteobacteria phyla with depth (78.7% in the shallow well above the ferricrete layer to 51.5% in the deep well below the ferricrete layer) and an increase with depth in the relative abundance of microorganisms belonging to the phyla Bacteroidetes, Acidobacteria, Chloroflexi, Planctomycetes, and Firmicutes (Fig. 5a). The decrease in Proteobacteria abundance and increase in overall alpha diversity with depth in the Cement Creek porewaters was primarily associated with a decrease in the abundance of *Gallionella* spp. (Fig. 5a), an Fe(II)-oxidizing, chemolithotropic bacteria. Although the abundance of *Gallionella* spp. decreased from 49% above the ferricrete layer to 21% below the ferricrete layer, the relative percent abundance of *Gallionella* was at least four times greater than any other genus at all sampling depths (Fig. 5a; Fig. S5 ). For example, percent increases in bacterial abundance associated with the *Ferrovum* (1%) and *WCHB1-32* (3.6%) genus were observed, but these abundances were minor compared to the ~29% decrease in the abundance of *Gallionella* with depth (Fig. S5). These changes in microbial abundance with depth in Cement Creek porewaters are consistent with those observed for the alpha (Fig. 3b) and beta diversity (Fig. 4a) measures.

For some phyla, different trends in microbial abundance were exhibited in the microcosm sediments compared to the porewaters. For example, Proteobacteria increased and Bacteroidetes decreased in relative abundance as a function of depth in the sediments (Fig. 5a).

#### Microbial community composition with depth: Mineral Creek

The percent relative abundance in five out of the six top phyla in the porewaters of Mineral Creek only varied a maximum of ±3% with depth (Fig. 5b). This is consistent with the relatively homogeneous water chemistry in the hyporheic zone of Mineral Creek (Fig. 2). While the abundance of phyla in the porewaters was relatively well-distributed, the microcosm sediments were characterized by a notable increase with depth in the abundance of microorganisms belonging to the Bacteroidetes phylum (8.5%–30.9%, Fig. 5b). The relative abundance of species in the genus *Pseudolabrys* decreased with depth in porewaters and sediments, and the relative abundance of genus *Actimicrobium* and *Flavobacterium* was higher in deep porewater and sediments compared to the shallow and intermediate samples (Fig. 5b; Fig. S6).

#### Differential abundance of attached and suspended microbial communities

As described in the methods, we used differential abundance analysis to test the null hypothesis that the taxa in the microcosm sediment samples were equally abundant compared to taxa present in the porewater samples, based on the log_2_ fold change [i.e., log_2_(mean abundance(microcosm sediments)/mean abundance(porewaters))]. The goal of this analysis was to evaluate the differential abundance of attached and suspended microbial communities in the different hyporheic zones of Mineral and Cement Creeks.

A greater number of taxa in Mineral Creek (~475) compared to Cement Creek (~90) were significantly differentially abundant (based on *P* < 0.001) in the microcosm sediments compared to the porewaters (see *y*-axis of Fig. S10 and S11 for the full distribution of *P*-values and log_2_ fold change values). This finding is consistent with the greater alpha diversity in Mineral Creek porewaters compared to Cement Creek porewaters (see the “Microbial diversity in hyporheic sediments and porewaters” section).

**Fig 6:**
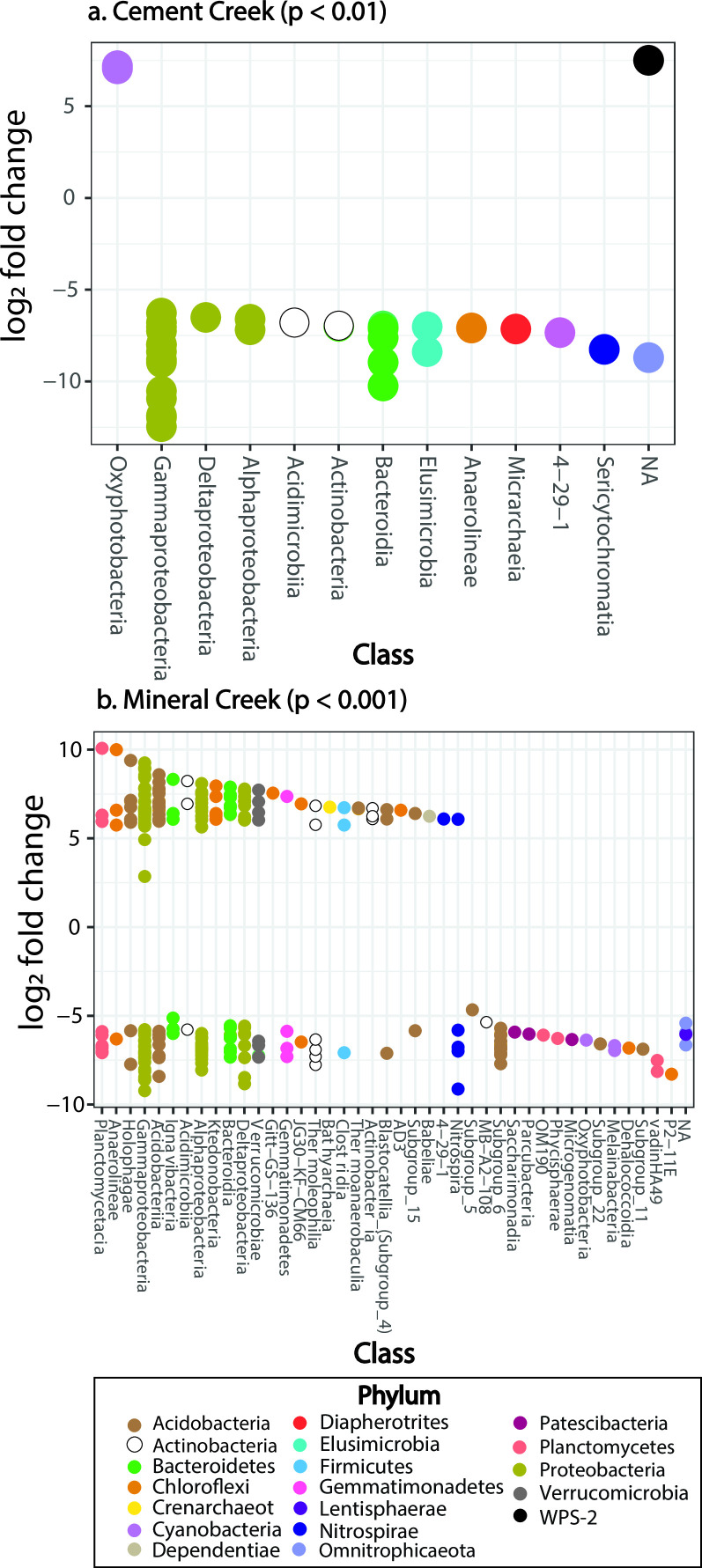
The class and phyla of the taxa that were identified as significantly different (*P* < 0.01 for Mineral Creek and Cement Creek, respectively) between the porewaters (taxa in negative log_2_ fold change space) and microcosm sediments (taxa in the positive log_2_ fold change space). Only the taxa with *P* < 0.001 are displayed here for Mineral Creek for visualization purposes, given that there were many more taxa that were significantly different between the microcosm sediments and porewaters at Mineral Creek. The most significant taxa classified by genus are presented in Figu. S12 and S13. No differentially abundant taxa were classified at the species level in Cement Creek and only three taxa that were differentially abundant in the microcosm sediments of Mineral Creek were classified at the species level. These included two Fe(III)-reducing species [*Geothrix fermentans;* (69) and *Geobacter psychrophilus;* (70)] and one facultatively anaerobic, sulfur-oxidizing, or nitrate-reducing autotroph [*Sulfuricella denitrificans;* (71)].

For Mineral Creek, the number of statistically significant taxa that were differentially abundant in the microcosm sediments was balanced with the number of taxa that were differentially abundant in the porewaters (Fig. S10). The taxa present in the porewaters but not in the microcosm sediments at Mineral Creek were from phyla including Nitrospirae, Planctomycetes, and Proteobacteria (Fig. 6b). Several of the significantly abundant taxa identified were from genera of nonmotile, biofilm-forming bacteria that oxidize ammonium [*VadinHA49;* (66)], nitrite [*Nitrospira;* (67)], and manganese [*Pedomicrobium;* (68)] (Fig. 6; Fig. S11). *Pseudolabrys*, a genus that exhibited a change in percent relative abundance with depth (Fig. 5b), was also differentially abundant in porewaters (Fig. S11).

For Cement Creek, only four significant taxa were differentially abundant in the microcosm sediments compared to the porewaters (Fig. S10). *Gallionella* and *Sulfuriferula* [an acidophilic, sulfur-oxidizing, autotroph; (72)] were identified as significantly differentially abundant in the porewaters of Cement Creek (Fig. S12), which is consistent with the identification of these as abundant genera in the heatmaps (Fig. 5a).

## DISCUSSION

### Geochemistry influences microbial diversity in hyporheic sediments and porewaters

**Fig 7:**
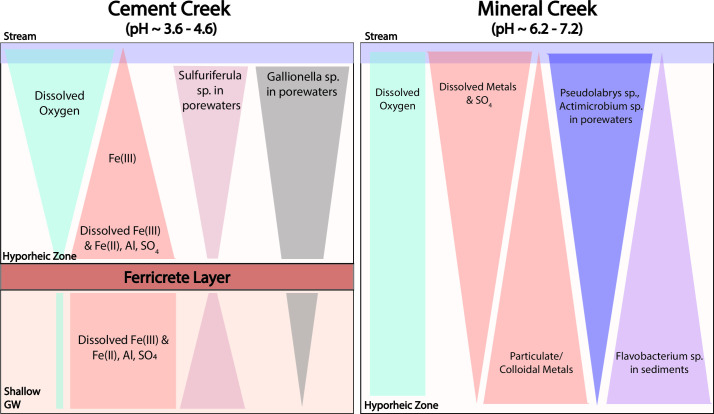
General trends in geochemistry and *Sulfuriferula*, *Gallionella*, *Pseudolabrys*, *Actimicrobium*, and *Flavobacterium* sp. as a function of depth in the stream and hyporheic zone of Cement and Mineral Creeks. The pH for each site corresponds to the range in pH across all sampling times and depths.

General trends in geochemistry and microbial abundance are summarized in Fig. 7. For Cement Creek, geochemistry differs above and below the ferricrete layer in Cement Creek and the microbial community data exhibits a depth-dependence. For example, the abundance of the Fe(II)-oxidizing species *Gallionella* decreases with depth relative to other low-abundance and specialized taxa. For Mineral Creek, dissolved oxygen concentrations are consistently high, metals are largely in the particulate or colloidal phase, and the microbial community composition differs between the porewaters and sediments.

Previous work in acid mine drainage streams has shown that microbial community diversity is largely driven by pH (73) and that microbial species richness is low at low pH given the small number of metabolically beneficial reactions available (74). The difference in porewater alpha diversity between Mineral and Cement Creeks is, thus, greatly influenced by differences in pH, where the pH in Mineral Creek was near neutral (pH ~6.5) across all sampling depths and the pH in Cement Creek was acidic (pH ~4) across all sampling depths.

Results from the principal component analysis suggest that geochemical and hydrological processes, in addition to pH, may influence microbial community structure at Cement Creek. The changes in alpha diversity, beta diversity, and hyporheic zone geochemistry as a function of depth in Cement Creek suggest that the ferricrete layer facilitates the development of microbial communities in the hyporheic zone that differ from specialized microbial communities in the deep portions of the streambed below the ferricrete layer. This finding supports our hypothesis for Cement Creek that the poor connection between the hyporheic zone and shallow groundwater, in combination with the acidic, metal-rich conditions, inhibits the physical mixing of suspended microorganisms in the water column and allows for rare and low abundance bacteria to dominate in the deep portions of the streambed. For Mineral Creek, the relationship between particulate metal concentrations and the beta diversity of the porewaters suggests that metal precipitates and colloids that form in the hyporheic zone, particularly in the deepest well, may allow for a unique phylogenetic community to develop in the porewaters.

### Groundwater-stream connectivity mediates hyporheic microbial composition

#### Well-connected groundwater-stream systems may facilitate the transport of nonmotile, metal-oxidizing bacteria throughout the hyporheic zone

The exchange of groundwater and surface water over a large hyporheic area (i.e., encompassing all three sampling wells) at Mineral Creek appears to have facilitated the homogenization of the porewater communities; however, the porewater and sediment microbiomes are distinct from one another (Fig. 4b and 6a). The greater number of differentially abundant taxa in both the microcosm sediments and porewaters for Mineral Creek samples indicates that the partitioning of microbial communities into a porewater microbiome and a sediment microbiome is not due to random chance. The differential abundance analysis also suggests that both the sediments and porewaters in the hyporheic zone of Mineral Creek foster many diverse species, and the community composition in the sediments is unique from the community composition in the porewaters. In comparison, Cement Creek microcosm sediments and porewaters have a lower number of highly specialized taxa that dominate.

Given that bacteria that can form biofilms were present in the porewater samples of Mineral Creek where total metal concentrations were high (see “Differential abundance of attached and suspended microbial communities” in the Results section), we hypothesize that nonmotile bacteria attach to metal-oxides that formed because of hyporheic mixing and move through hyporheic pore spaces. Furthermore, biofilms were observed on the *in situ* microcosm sediment bags as well as the streambed surface at Mineral Creek. In cases where water velocities are sufficiently high, cells can be dislodged and transported away from the surfaces or biofilms they were adhered to (75). Mechanical transport of cells in this manner depends on factors such as cell size, biofilm size, shear stress, and flow velocity and can be estimated by the Péclet number (or the ratio of the rate of advective transport to diffusive transport) (76). Although the velocity of surface water infiltrating into the hyporheic zone was high in Mineral Creek (~0.7 m/d or 8.1 × 10^−6^ m/s) compared to Cement Creek (<0.16 m/d or <1.9 × 10^−6^ m/s), these velocities are several orders of magnitude lower than the range of velocities previously shown to result in cell detachment in experimental studies (75, 77) and mountainous streams (78). Thus, a more likely transport mechanism is that bacteria (with individual cell sizes of 0.2–2 µm) remain attached to particles and colloids as biofilms (with biofilm aggregates on the order of 10 s of microns in size) that are transported through hyporheic pore spaces of this gravelly streambed (millimeter scale pores) and eventually discharged to the stream due to hyporheic mixing.

The physical transport of microorganisms at Mineral Creek facilitated by metal-oxide formation in the hyporheic zone could serve several important ecosystem functions. First, the presence of bacteria from the Nitrospirae and Planctomycetes phyla could be important for nutrient cycling in the hyporheic zone of Mineral Creek; however, nitrate and organic carbon concentrations are relatively low in Mineral Creek (Fig. 2b) and these phyla contain a diverse group of microorganisms with many functions. Additional nutrient sampling, functional assays, and metagenomics analysis could help elucidate the function and activity of these microbial communities. Second, nonmotile bacteria attached to metal-oxide particles may help with cell dispersal to sediment microbial communities that may otherwise be inaccessible (29). Finally, the transport of nonmotile forms of biofilm-forming, metal-oxidizing bacteria such as *Pedomicrobium* (79) attached to particles could contribute to the natural attenuation of dissolved manganese from upwelling groundwater via the precipitation of manganite [MnO(OH); (80)], which was predicted to precipitate according to geochemical calculations in (37).

#### Fe(II)-oxidizers dominate in acidic, poorly connected groundwater-stream systems and may limit hyporheic exchange

Given the change in both the relative abundance for certain genera and redox chemistry with depth at Cement Creek (Fig. 7), we propose that the ferricrete layer not only limits groundwater-surface water exchange but also creates a microbial diversity barrier beneath the streambed. In this iron-rich system, *Gallionella* sp. persist throughout the subsurface of Cement Creek but decrease in relative abundance with depth. Another Fe(II)-oxidizer, *Sideroxydans* sp.*,* specifically *Sideroxydans lithotrophicus,* was one of the most relatively abundant species at the ferricrete layer (Fig. S13). At and below the ferricrete layer, a more diverse array of low-abundance species with diverse microbial metabolisms existed likely because of higher dissolved metal concentrations (Fig. 2e), lower dissolved oxygen concentrations (Fig. 2g), and potentially a hydrologic connection to the shallow groundwater reservoir in the adjacent iron fen that had alpha diversity comparable to the deepest porewater samples (Fig. 3a).

The microbial community composition at Cement Creek is consistent with other acid mine drainage systems, where electron donor/acceptor gradients and low pH create conditions favorable for highly specialized and niche microbial metabolic functions and taxonomies (81–83). Yet, differences in microbial community composition above and below the ferricrete layer were present even though the pH did not vary notably with depth and remained between pH 3.6 and 4.6 (Fig. 7). As pH increases from extremely acidic (pH < 2) to moderately acidic conditions (pH 4–6) such as at Cement Creek, abiotic chemical Fe(II) oxidation becomes increasingly favorable over microbially mediated Fe(II) oxidation (84), and the relative contribution of microbial versus abiotic mediation of Fe(II) oxidation is not well understood. Although *Gallionella* spp. are known to typically grow at pH ranging from 5 to 7.5 (85, 86), microaerobic *Gallionella* spp. and other Fe(II)-oxidizing bacteria were more abundant than other taxa throughout the Cement Creek hyporheic zone. The high abundance of *Gallionella* spp. is consistent with findings from reference (87), which found that *Gallionella* spp. had the greatest relative abundance in mine-impacted sediments at pH ~4.4 and exhibited greater metal and acid tolerance than expected. To our knowledge, this study is one of the few since (87) to highlight the importance of *Gallionella* spp. and other Fe(II)-oxidizing bacteria in moderately acidic (pH 4–6), metal-rich environments.

The presence of a distinct sequence in microbial community composition and physiological capabilities with depth is consistent with findings for other low-permeability sediments (88). However, in acid-mine drainage systems such as Cement Creek, the distribution of lithoautotrophic Fe(II)-oxidizers such as *Gallionella* sp. and *Sideroxydans lithotrophicus* below the streambed may not only be a result of low-permeability sediments but also a contributing factor to the permeability of the sediments. These species could facilitate a positive feedback loop where the microbially mediated precipitation of amorphous iron oxides clog hyporheic pore spaces and increasingly separate the microbial communities located above the ferricrete layer from those below similar to the impacts of bio-clogging documented in other hyporheic systems (89, 90). Furthermore, *Sideroxydans lithotrophicus* were found to enhance Fe(II) oxidation at pH 4–6 in the presence of fen-derived humic acids and resulted in nano-sized Fe(III)oxyhydroxide precipitates (91, 92). Thus, organics derived from the iron fen adjacent to Cement Creek could play an interesting yet unexplored role in the formation of the ferricrete layer and the role of Fe(II)-oxidizing microorganisms in the hyporheic zone. These findings for Cement Creek may be applicable to other hyporheic zones where the formation of clogging layers composed of oxidized crusts results from iron-rich discharge to streams (93, 94).

### Conclusions

By investigating two streams located downstream of abandoned mine drainages in the Animas River headwaters, we identified a link between pH and metal-oxide precipitation, groundwater-surface water connectivity, and hyporheic microbiome composition. In systems such as Mineral Creek where metal-rich groundwater and oxygenated, metal-poor stream water frequently exchanges, hyporheic mixing led to the formation of metal-oxide colloids and particles in porewaters. In this well-connected hyporheic system, the hydrogeochemical conditions support high alpha diversity and phylogenetic similarities in porewaters were correlated with particulate metal concentrations. Furthermore, species that can be nonmotile and biofilm-forming, including *Pedomicrobium*, *VadinHA49,* and *Nitrospira,* were differentially abundant in porewaters. We hypothesize that the transport of manganese-, ammonium-, and nitrite-oxidizing microorganisms attached to colloids and particles in the hyporheic pore spaces may enhance the natural attenuation of reduced metals in Mineral Creek. Future research could test this hypothesis by analyzing biofilms and particles collected on filters with scanning electron microscopy.

In systems such as Cement Creek where the hyporheic zone is small and groundwater-surface water exchange is limited due to abundant iron-oxide precipitation, overall microbial diversity is relatively low compared to Mineral Creek due to the lower pH (~4), higher dissolved metal concentrations, and limited hyporheic mixing. Fe(II)-oxidizing bacteria such as *Gallionella* were prevalent in the hyporheic zone of Cement Creek compared to other microorganisms. The relative contribution and community composition of Fe(II)-oxidizing species in systems with moderately low pH (4–6) but not extremely acidic pH (<2) remains understudied. Despite the higher relative abundance of *Gallionella* compared to other niche species, the abundance of *Gallionella* below the ferricrete was lower than the abundance above the ferricrete in the hyporheic zone. This suggests that limited groundwater-stream exchange at Cement Creek impacts and is impacted by microbially mediated iron-oxide precipitation. Furthermore, the correlation between beta diversity and environmental variables that exhibited strong chemical gradients (i.e., dissolved O_2_, Mn, Al, and Fe) suggests that the ferricrete barrier facilitates not only redox stratification but also the development of niche microbiome assemblies. Complementary studies that could further test the link between microbiome structure and hyporheic mixing processes include RNA analyses to evaluate active metabolisms that influence metal chemistry (95), functional assays or column studies to test microbial behavior under controlled hyporheic conditions (96–98), or microscopy with Fluorescence *in situ* hybridization (FISH) staining to observe bacteria attached to metal-oxide colloids.

The findings presented in this study have potential implications for the cycling of metals and nutrients in acid-mine drainage streams. In streams such as Mineral Creek, large, well-mixed hyporheic zones may facilitate the transport of diverse microorganisms through hyporheic pore spaces, which could inhibit the export of dissolved metals downstream. Low-pH streams where hyporheic exchange is inhibited by metal-oxide precipitation, such as Cement Creek, are characterized by abundant putative iron-oxidizing bacteria and low phylogenetic diversity. The prevalence of iron-oxidizing bacteria likely contributes to additional clogging of hyporheic pore spaces, which lowers fluid residence times and leads to the accumulation of toxic metals in the hyporheic zone.

## Data Availability

Data generated or analyzed during this study are included in this published article and its supplementary information file. Additional hydrological data can be found in the HydroShare database at http://www.hydroshare.org/resource/c9ef6ecde25640d4bd4c7a9c50575016. DNA sequences were submitted to the National Center for Biotechnology Information (NCBI) Sequence Read Archive (SRA) database under BioProject number PRJNA952531. The sequence data can be accessed at https://www.ncbi.nlm.nih.gov/sra/?term=PRJNA952531.

## SUPPLEMENTAL MATERIAL

Supplemental material aem.01987-23-s0001.docx All supplemental text, figures, and tables included.

ASM does not own the copyrights to Supplemental Material that may be linked to, or accessed through, an article. The authors have granted ASM a non-exclusive, world-wide license to publish the Supplemental Material files. Please contact the corresponding author directly for reuse.
