# Supplementary material for: Metal-oxide precipitation influences microbiome structure in hyporheic zones receiving acid rock drainage
Source: Appl Environ Microbiol. 2024 Feb 23;90(3):e01987-23. doi: 10.1128/aem.01987-23 (PMC10952486; doi:10.1128/aem.01987-23)
Supplement: Supplemental material — All supplemental text, figures, and tables included. [file aem.01987-23-s0001.docx]

**Supporting Information for**

**Metal-oxide precipitation influences microbiome structure in hyporheic porewaters of streams receiving acid rock drainage**

**Introduction**

The supplementary information below includes a description of the 16S rRNA gene sequencing protocol and differential abundance analysis using DESeq2. Also included are table summaries of the sediment sample registrations, quality control, and processing, and PERMANOVA calculations (Tables S1-S4). We also include rarefaction curves (Figure S1), alpha diversity comparison for rarefied and non-rarefied samples (Figure S2), beta diversity of streambed and microcosm sediment samples (Figure S3), beta diversity CAP ordination calculated from unweighted unifrac distances (Figure S4), heatmaps classified to the genus level for Cement Creek and Mineral Creek porewaters and sediments (Figures S5-S6), heatmap for sample controls (Figures S7), heat maps for stream samples (Figure S8), heat maps classified to the species level for Cement and Mineral Creek porewaters and sediments (Figure S9), p-value distributions corresponding to the differential abundance analysis (Figure S10), volcano plots for the differential abundance analysis (Figure S11), differentially abundant bacteria in sediments and porewaters classified by Phylum and Genus for Mineral and Cement Creeks (Figure S12-13), and beta diversity for all samples (streambed, porewaters, microcosms, and groundwater; Figure S14).

**16S rRNA Gene Analyses – Method Description**

Total community DNA was extracted from streambed and microcosm sediments and filters using the ZymoBIOMICS DNA Miniprep Kit (Cat No. D4300) and the binding preparation protocol for soil samples described in the kit. Extraction yields for each sample are reported in Table S2. Extraction and sequencing methods were completed following the protocol in (Kraus et al., 2018). In summary, archaeal and bacterial 16S rRNA gene libraries were amplified with a 30-cycle polymerase chain reaction (PCR) using primer set 515F/926R, which targets the V4 and V5 hypervariable regions of the 16S rRNA gene between position 515 and 926 (*Escherichia coli* numbering) (Kraus et al., 2018; Parada et al., 2016). The forward primer 515F-Y (GTA AAA CGA CGG CCA G CCG TGY CAG CMG CCG CGG TAA-3’) contains the M13 forward primer (GTA AAA CGA CGG CCA G) fused to the gene-specific forward primer (CCG TGY CAG CMG CCG CGG TAA), while the reverse primer 926R (5’-CCG YCA ATT YMT TTR AGT TT- 3’) was unmodified from (Parada et al., 2016). Targeting this region produces a fragment of ~400 base pair (bp) for the Bacteria and Archaea 16S rRNA gene. Assurance of quality control was ensured by performing DNA extractions on field replicates (Table S3) and three controls (i.e. nuclease-free water), including a negative and a positive (ZymoBIOMICS Microbial Community Standard, Cat No. D6305) control in the PCR amplification process. Extractant concentrations were measured using fluorometry (Qubit 4 Fluorometer) and all PCR amplification products were verified using a 1% agarose gel with 50 bp and 1 kilobase pair ladders, which are references for estimating the size and concentration of the extracted DNA fragments. The final pooled library was submitted for high throughput sequencing on the Illumina MiSeq platform using the PE250 V2 chemistry method (Illumina, San Diego, CA, USA) at the Duke Center for Genomic and Computational Biology.

**Differential Abundance Analysis – Method Description**

Differential abundance analysis was performed on 16S rRNA gene data to test the null hypothesis that the abundance of taxa in the microcosm sediment samples were similar to the abundance in the porewater samples. In our analysis, microcosm sediment species abundances were the reference condition and porewater species abundances were the comparison condition. The analysis was conducted using the DESeq2 package (Love et al. 2014), which takes a normalized count matrix (i.e. number of times a sequence associated with a 16S rRNA gene appears in each sample) and models the abundance of a feature in a given sample with a negative binomial distribution. A negative binomial distribution is a more representative model for the sequence data because the read counts for a sample are often over-dispersed (i.e. the sample variance exceeds the sample mean), whereas the variance and mean in a Poisson distribution are the same. Three samples were identified as outliers using principal components analysis and Cook’s cutoff distances and removed from the differential abundance analysis (Table S3).

After outlier identification and removal, seven porewater and five microcosm sediment samples remained for Mineral Creek and five porewater and four microcosm sediment samples remained for Cement Creek. Pre-filtering was conducted to remove taxa with low read counts, where only taxa with read counts greater than or equal to three in more than three samples were kept in the analysis. In the analysis, we used a Wald test, or an assessment of constraints on statistical parameters on weighted, quantitative distances, to determine the significance of coefficients in a negative binomial generalized linear model and a parametric regression to fit dispersion estimates (i.e. log(dispersion)/log(mean of normalized counts)). Differential abundances characterized by a log fold change (lfc) ≥ 2 and a corrected p-value ≤ 0.01 were considered significantly different and the null hypothesis that microcosm sediments and porewaters have similar species abundance could be rejected. The ratio method was used to estimate size factors, which represent the median value of the ratios of the geometric mean of counts in one sample to the geometric mean of counts across all samples (Anders and Huber 2010). The purpose of this scaling technique is to make taxa counts from different samples that may have been sequenced to different depths comparable. Further, we used the lfcShrink function to reduce the effect size of one sample on the estimated fold change and provide more conservative estimates of significant differential abundances (Love et al. 2014). lfcShrink is particularly useful when sample sizes are small.

| **Table S1.** Sediment samples registered with SESAR at geosamples.org. | | | |
| --- | --- | --- | --- |
| **Sample Name** | **Stream System** | **Parent IGSN** | **IGSN** |
| MC LB 0 – 10 cm | Mineral Creek | IENEH0040 | IENEH0045 |
| MC LB 11 – 20cm | Mineral Creek | IENEH0040 | IENEH0046 |
| MC LB 21 – 30 cm | Mineral Creek | IENEH0040 | IENEH0047 |
| MC LB 31 – 40 cm | Mineral Creek | IENEH0040 | IENEH0048 |
| MC LB 41 – 50 cm | Mineral Creek | IENEH0040 | IENEH0049 |
| MC LB 51 – 60 cm | Mineral Creek | IENEH0040 | IENEH004A |
| MC LB 61 – 67 cm | Mineral Creek | IENEH0040 | IENEH004B |
| MC LB 11 – 20 cm < 2mm | Mineral Creek | IENEH0040 | IENEH004C |
| MC LB 31 – 40 cm <2mm | Mineral Creek | IENEH0040 | IENEH004D |
| MC LB 61 – 67 cm <2mm | Mineral Creek | IENEH0040 | IENEH004E |
| MC LB Streambed 0 – 4 cm | Mineral Creek | IENEH0043 | IENEH004F |
| MC LB Streambed 0 – 4 cm < 2mm | Mineral Creek | IENEH0043 | IENEH004G |
| CC LB Streambed 0 – 4 cm < 2mm | Cement Creek | IENEH0042 | IENEH004H |
| CC LB Streambed 0 – 4 cm | Cement Creek | IENEH0042 | IENEH004I |
| CC LB 0 – 10 cm | Cement Creek | IENEH0041 | IENEH004J |
| CC LB 11 – 20 cm | Cement Creek | IENEH0041 | IENEH004K |
| CC LB 0 – 10 cm < 2mm | Cement Creek | IENEH0041 | IENEH004L |
| CC LB 11 – 20 cm < 2mm | Cement Creek | IENEH0041 | IENEH004M |
| CC Iron Fen 0 – 10 cm | Cement Creek | IENEH0044 | IENEH004N |
| CC Iron Fen 11 – 20 cm | Cement Creek | IENEH0044 | IENEH004O |

| **Table S2. DNA extraction yields measured using a Qubit Fluorometer** | | | | | | | | |
| --- | --- | --- | --- | --- | --- | --- | --- | --- |
| **System** | **Lab ID** | **Accession #^a^** | **Sample ID** | **Type** | **Depth Interval (cm)*** | **Sample Date** | **Extraction Matrix** | **DNA Biomass (ng/𝝻L)** |
| QA/QC | Control |  | Blank sample with DNA extraction reagents | Control |  |  | Blank | < 0.005 |
|  | Ctl2 |  | Control 2 |  |  |  | Blank | 0.006 |
|  | Ctl3 |  | Control 3 |  |  |  | Blank | 0.005 |
| Cement Creek | CC1 | SAMN34075051 | CC at PVC 0 - 10cm 190802 | Streambank Sediment | 0-10 | 8/2/2019 | Sediment | 0.020 |
|  | CC2 | SAMN34075052 | CC at PVC 10 - 20cm 190802 |  | 10-20 | 8/2/2019 | Sediment | 0.013 |
|  | CC3 | SAMN34075053 | CC 28 cm 190920 bag 1 | Microcosm Sediment | 18-28 | 9/20/2019 | Sediment | 0.006 |
|  | CC4 | SAMN34075054 | CC 44 cm 190920 bag1 |  | 34-44 | 9/20/2019 | Sediment | 0.008 |
|  | CC4-2 | SAMN34075055 | CC 44 cm 190920 bag2 |  | 34-44 | 9/20/2019 | Sediment | 0.008 |
|  | CC5 | SAMN34075056 | CC-58 cm 190920 bag1 |  | 48-58 | 9/20/2019 | Sediment | 0.009 |
|  | CC5-2 | SAMN34075057 | CC-58 cm 190920 bag2 |  | 48-58 | 9/20/2019 | Sediment | 0.008 |
|  | CCS1 | SAMN34075058 | CC Stream 190731 | Stream | 0 | 7/31/2019 | Filter | 0.019 |
|  | CCS2 | SAMN34075059 | CC Stream 190920 |  | 0 | 9/20/2019 | Filter | 0.014 |
|  | CC1w | SAMN34075060 | CC 28cm 190731 WC | Porewater | 18-28 | 7/31/2019 | Filter | 0.015 |
|  | CC4w | SAMN34075061 | CC 28 cm 190920 WC |  | 18-28 | 9/20/2019 | Filter | 0.043 |
|  | CC2w | SAMN34075062 | CC 44cm 190731 WC |  | 34-44 | 7/31/2019 | Filter | 0.008 |
|  | CC5w | SAMN34075063 | CC 44 cm 190920 WC |  | 34-44 | 9/20/2019 | Filter | 0.021 |
|  | CC3w | SAMN34075064 | CC 58cm 190731 WC |  | 48-58 | 7/31/2019 | Filter | 0.008 |
|  | CC6w | SAMN34075065 | CC-58 cm 190920 WC |  | 48-58 | 9/20/2019 | Filter | 0.009 |
|  | F1 | SAMN34075066 | CC in Fen d/s PG 0 -10 | Fen Soil (data not presented) | 0-10 | 6/19/2022 | Sediment | 0.038 |
|  | F2 | SAMN34075067 | CC in Fen d/s PG 10 -20 |  | 10-20 | 6/19/2022 | Sediment | 0.078 |
|  | S2 | SAMN34075068 | CC Seep 18:30 | Seep (data not presented) | 0 | 6/22/2019 | Sediment | 0.056 |
|  | S1 | SAMN34075069 | CC Seep 8:30am |  | 0 | 6/22/2019 | Sediment | 0.095 |
|  | CCW9-2 | SAMN34075070 | CC Well 9 190731 WC | Groundwater |  | 7/31/2019 | Filter | 0.233 |
|  | CCW9 | SAMN34075071 | CC Well 9 190920 WC |  |  | 9/20/2019 | Filter | 1.890 |
|  |  |  |  |  |  |  |  |  |
| **Table S2. DNA extraction yields measured using a Qubit Fluorometer** | | | | | | | | |
| **System** | **Lab ID** | **Accession #^a^** | **Sample ID** | **Type** | **Depth Interval (cm)*** | **Sample Date** | **Extraction Matrix** | **DNA Biomass (ng/𝝻L)** |
| Mineral Creek | MC1 | SAMN34075072 | MC LB Fen Sediment 10-20 cm | Streambank Sediment | 10-20 | 6/21/2019 | Sediment | 2.750 |
|  | MC2 | SAMN34075073 | MC LB Fen Sediment 30-40 cm |  | 30-40 | 6/21/2019 | Sediment | 0.029 |
|  | MC3 | SAMN34075074 | MC LB Fen Sediment 60-68 cm |  | 60-68 | 6/21/2019 | Sediment | 0.056 |
|  | MC4 | SAMN34075075 | MC 20cm 190927 bag1 | Microcosm Sediment | 10-20 | 9/27/2019 | Sediment | 5.700 |
|  | MC4-2 | SAMN34075076 | MC 20cm 190927 bag2 |  | 10-20 | 9/27/2019 | Sediment | 5.700 |
|  | MC4-3 | SAMN34075077 | MC 20cm 190927 bag3 |  | 10-20 | 9/27/2019 | Sediment | 5.200 |
|  | MC5 | SAMN34075078 | MC 40cm 190927 bag1 |  | 30-40 | 9/27/2019 | Sediment | 0.246 |
|  | MC6 | SAMN34075079 | MC 68cm 190927 bag1 |  | 58-68 | 9/27/2019 | Sediment | 0.028 |
|  | MCS2 | SAMN34075080 | MC Stream 190927 | Stream | 0 | 9/27/2019 | Filter | 0.313 |
|  | MC1w | SAMN34075081 | MC 20cm 190621 WC | Porewater | 10-20 | 6/21/2019 | Filter | 0.132 |
|  | MC4w | SAMN34075082 | MC 20cm 190927 WC |  | 10-20 | 9/27/2019 | Filter | 0.079 |
|  | MC2w | SAMN34075083 | MC 40cm 190620 WC |  | 30-40 | 6/20/2019 | Filter | 0.321 |
|  | MC2w-9 | SAMN34075084 | MC 40cm 190620 WC 9am |  | 30-40 | 6/20/2019 | Filter | 0.163 |
|  | MC5w | SAMN34075085 | MC 40cm 190927 WC |  | 30-40 | 9/27/2019 | Filter | 0.053 |
|  | MC3w | SAMN34075086 | MC 68cm 190621 WC |  | 58-68 | 6/21/2019 | Filter | 0.095 |
|  | MC6w | SAMN34075087 | MC 68 cm 190927 WC |  | 58-68 | 9/27/2019 | Filter | 0.205 |
|  | MC6w2 | SAMN34075088 | MC 68 cm 190927 WC Rep2 |  | 58-68 | 9/27/2019 | Filter | 0.166 |
|  | MC6w3 | SAMN34075089 | MC 68 cm 190927 WC Rep3 |  | 58-68 | 9/27/2019 | Filter | 0.217 |
| *For the streambank sediments, the depth interval corresponds to the interval over which composite sediment samples were collected. For the porewater and microcosm sediments, the depth interval corresponds to the screened interval of the in situ microcosm wells. | | | | | | | | |
| a. All samples were submited to National Center for Biotechnology Information (NCBI) Sequence Read Archive (SRA) database under BioProject # PRJNA952531 and can be accessed here: https://www.ncbi.nlm.nih.gov/sra/PRJNA952531. | | | | | | | | |

| **Table S3.** Samples that underwent replicate DNA extractions for rRNA gene analysis. | | | | |
| --- | --- | --- | --- | --- |
| **Stream** | **Sample Type** | **Depth (cm)** | **Collection Date** | **# of Replicates** |
| Cement Creek | Sediment | 44 | 9/20/2019 | 2 |
| Cement Creek | Sediment | 58 | 9/20/2019 | 2 |
| Mineral Creek | Sediment | 20 | 9/27/2019 | 3 |
| Mineral Creek | Porewater (filter paper) | 68 | 9/27/2019 | 3 |

| **Table S4.** Samples removed from downstream analyses in R. | | | | |
| --- | --- | --- | --- | --- |
| **Stream** | **Sample Type** | **Depth (cm)** | **Collection Date** | **Reason for removal** |
| Cement Creek | Microcosm Sediment | 44 | 9/20/2019 | Outlier identified using PCA and Cooks Cutoff Distances in DESeq2 |
| Cement Creek | Porewater | 58 | 7/31/2019 | Outlier identified using PCA and Cooks Cutoff Distances in DESeq2 |
| Mineral Creek | Porewater | 20 | 9/27/2019 | Low sequence sample size (6002 counts) and outlier identified in calculation of diversity measures (see Figure S3) |

**Figure S1.** Rarefaction curves generated in phyloseq for all groundwater, porewater, and sediment samples. The Mineral Creek porewater sample (MC4w) with a low sequence sample size (6002 counts) was removed prior to downstream analyses in R.

**Figure S2.** Comparison of alpha diversity estimates on rarefied (top) and non-rarefied (bottom) datasets for all samples prior to any sample removal.


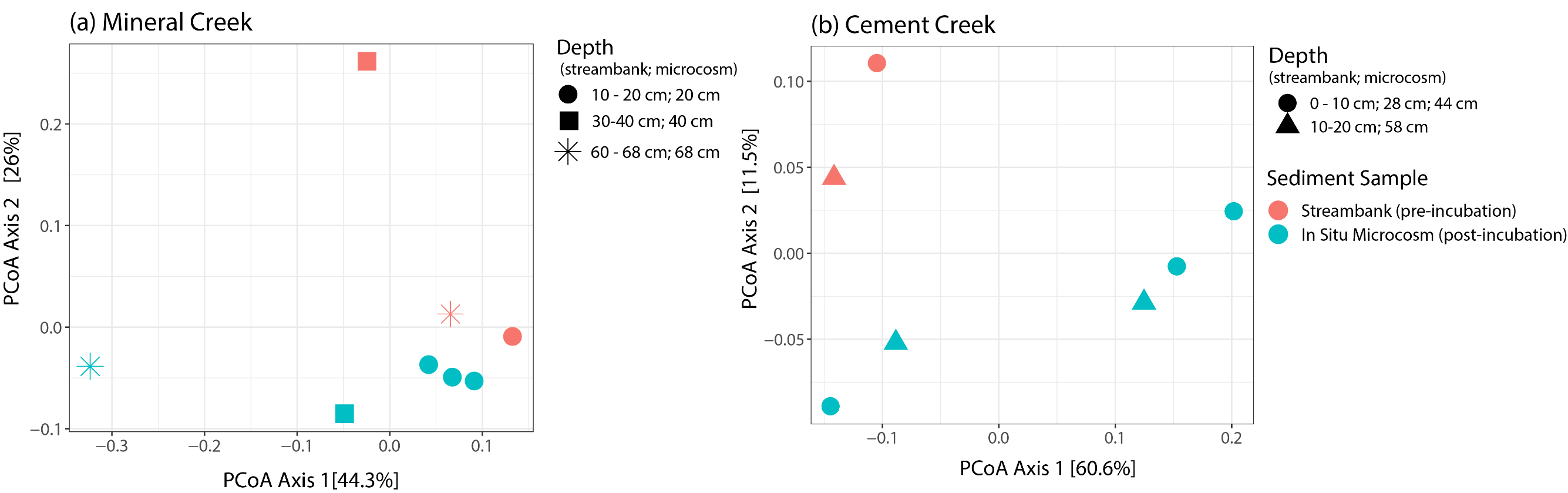


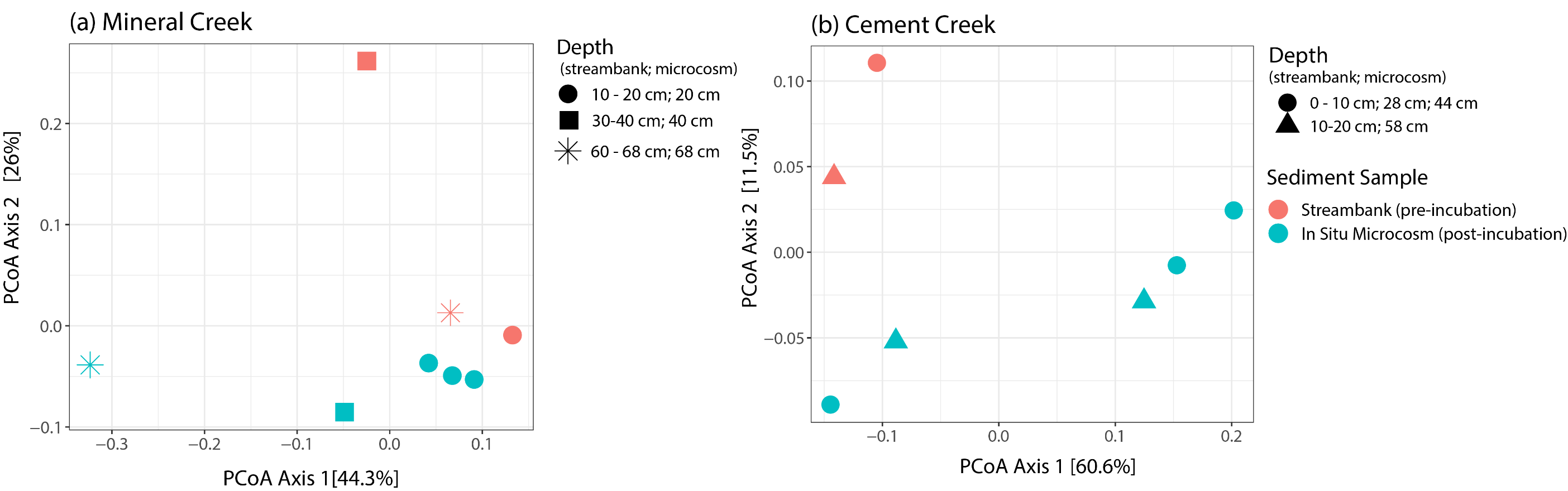


**Figure S3.** Beta diversity of streambank samples and in situ microcosm sediment samples collected after incubation in the well clusters. Depth symbology corresponds the streambank sample to the depth interval the sample was deployed at in the *in-situ* microcosm bags for incubation.


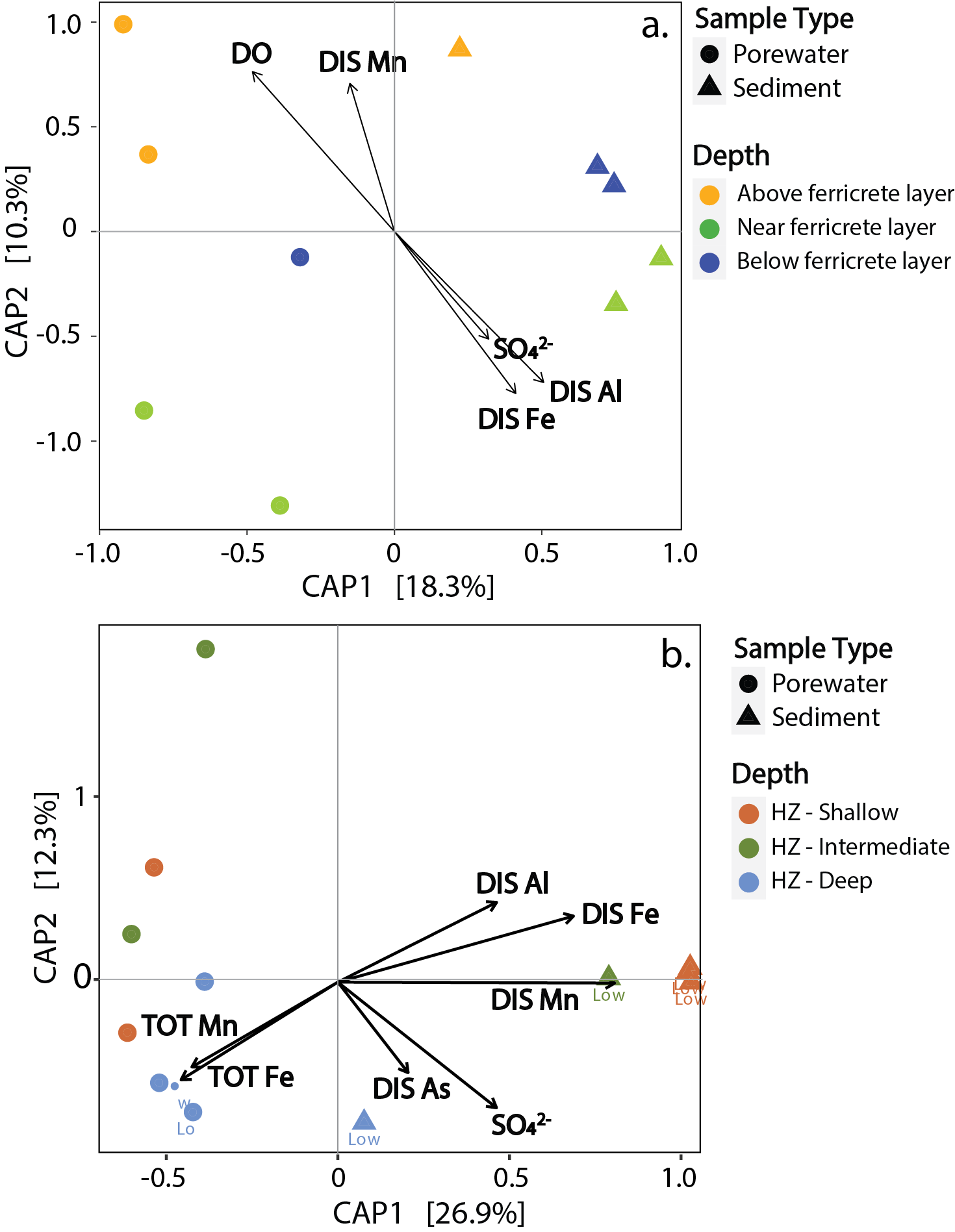


**Figure S4**. Beta-diversity calculations for microbial communities in (a) Cement Creek and (b) Mineral Creek for porewater and microcosm sediment samples. Distances between plotted points are related to their phylogenetic dissimilarities and estimated using unweighted Unifrac distancing. TOC = total organic carbon; DO = dissolved oxygen; TOT = total concentration; DIS = dissolved concentration.


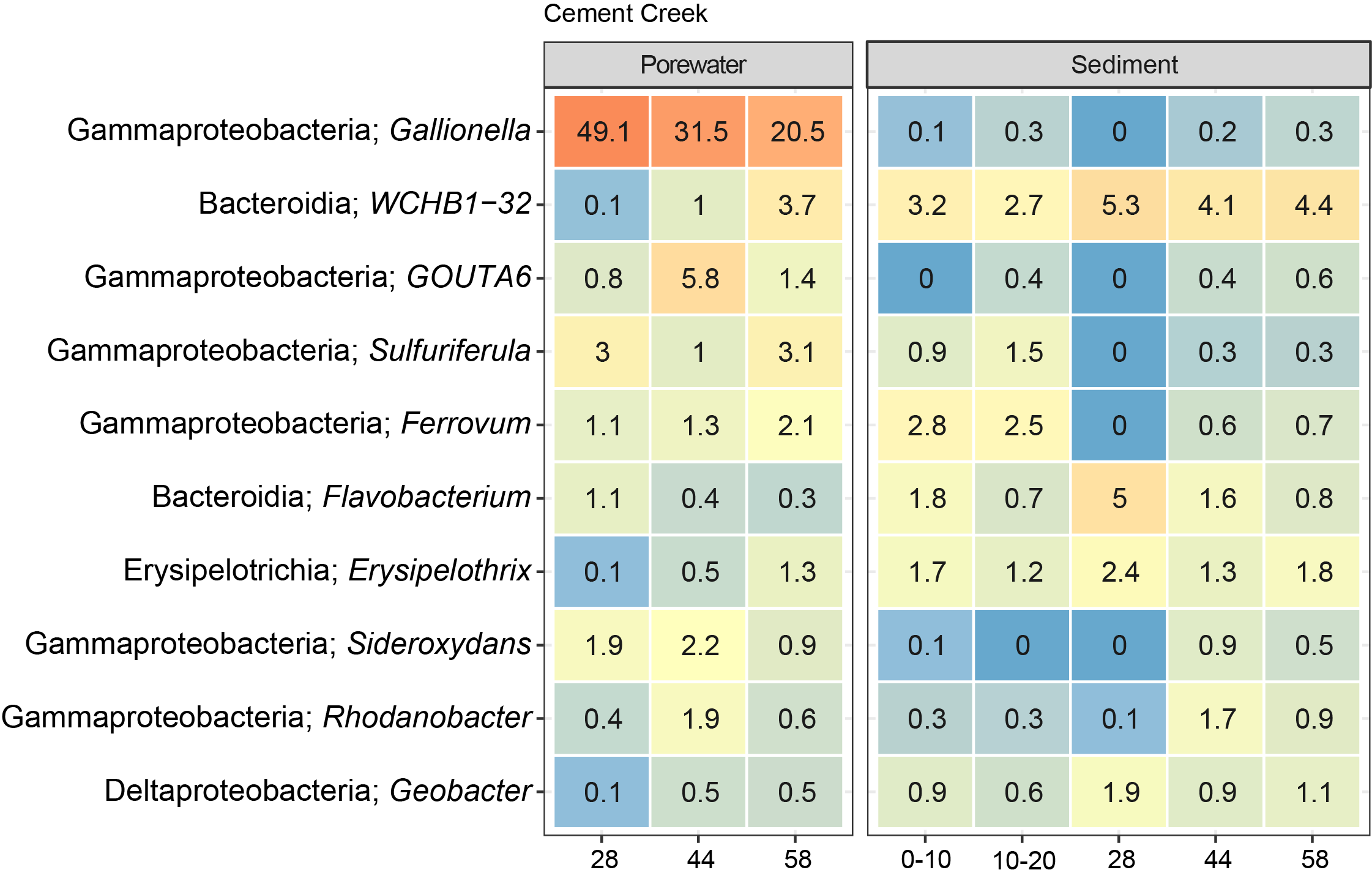


**Figure S5.** Heatmap of the percent relative abundance of the amplicon sequence variants (ASV) categorized by class and genus from Cement Creek. Sediment samples labeled with depth intervals “0-10” and “10-20” represent streambed sediments and samples labeled with depth intervals “28”, “44”, or “58” represent microcosm sediments.

**
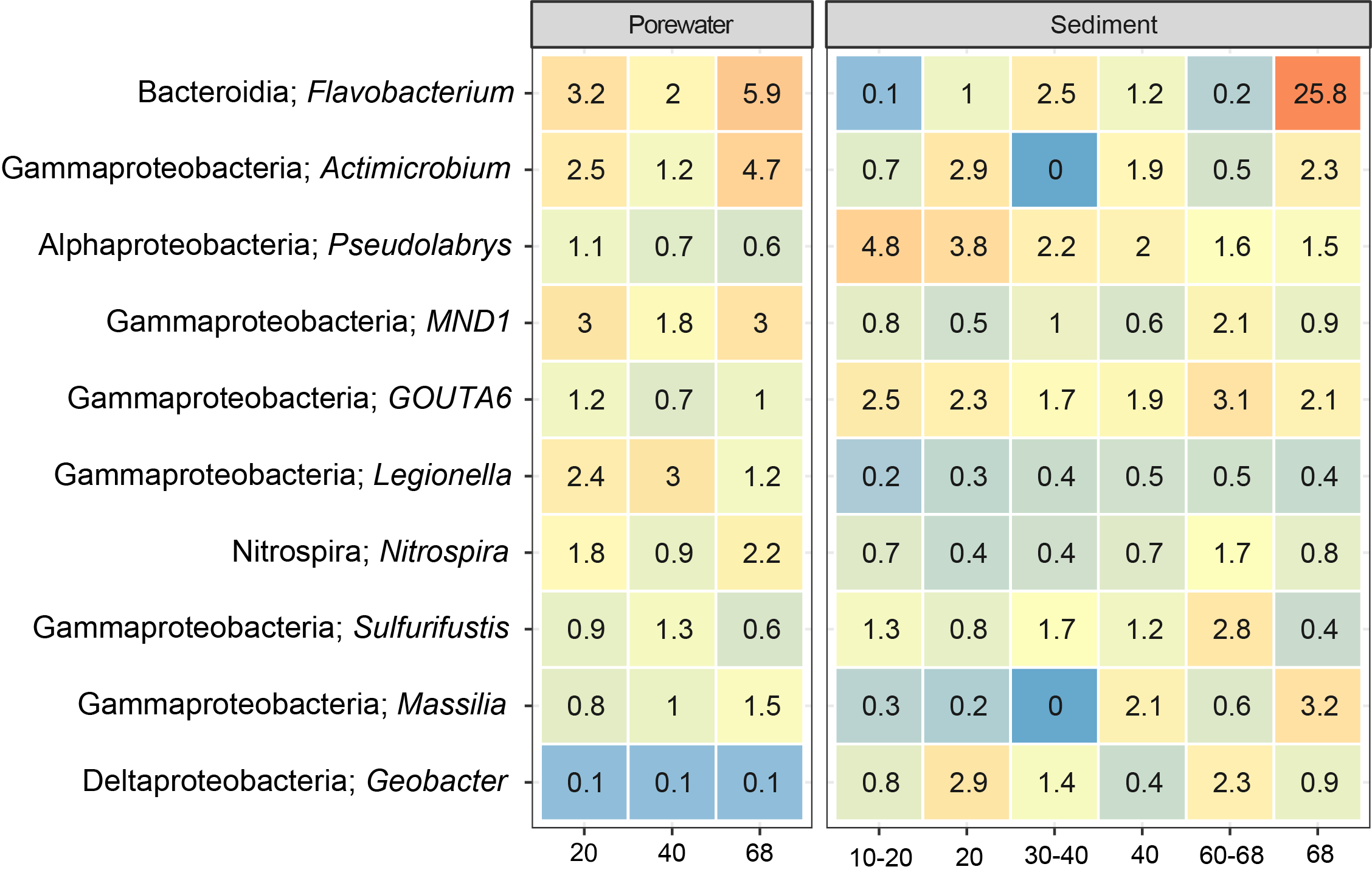
**

**Figure S6.** Heatmap of the percent relative abundance of the amplicon sequence variants (ASV) categorized by class and genus from Mineral Creek. Sediment samples labeled with depth intervals “10-20”, “30-40”, or “60-68” represent streambed sediments and samples labeled with depth intervals “20”, “40”, or “68” represent microcosm sediments.


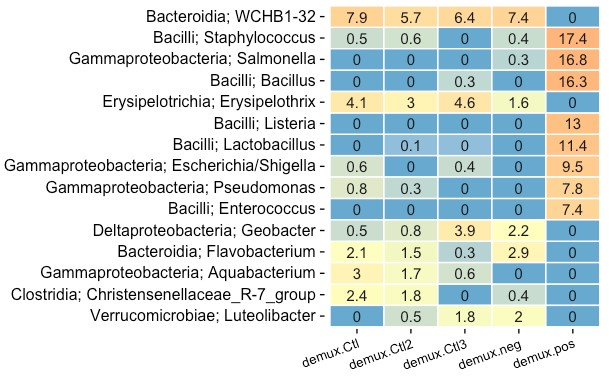


**Figure S7.** Heatmap of the percent relative abundance of the amplicon sequence variants (ASV) categorized by phyla and genus that were measured on negative extraction controls (demux.Ctl, demux.Ctl2, demux.Ctl3), and a negative and positive PCR control (ZymoBIOMICS Microbial Community Standard).


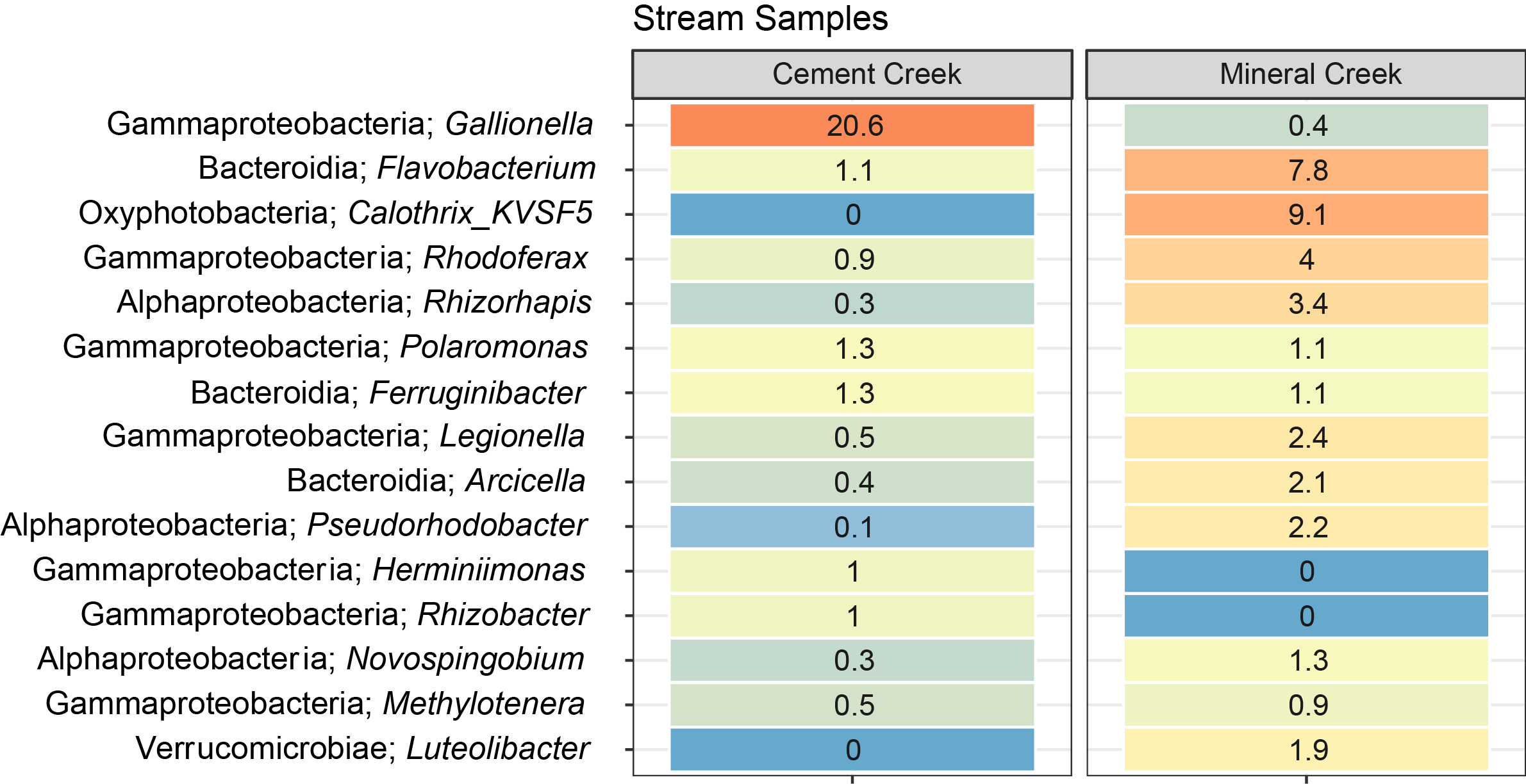

**Figure S8.** Percent read abundances for samples collected from the stream of Cement Creek and Mineral Creek faceted by class and genus (top) and phylum and class (bottom).

**Figure S9.** Heatmap of percent read abundances of the amplicon sequence variants (ASV) categorized by genus and species for Cement and Mineral Creeks. Cement Creek sediment samples labeled with depth intervals “0-10” and “10-20” represent streambed sediments and samples labeled with depth intervals “28”, “44”, or “58” represent microcosm sediments. Mineral Creek sediment samples labeled with depth intervals “10-20”, “30-40”, or “60-68” represent streambed sediments and samples labeled with depth intervals “20”, “40”, or “68” represent microcosm sediments.


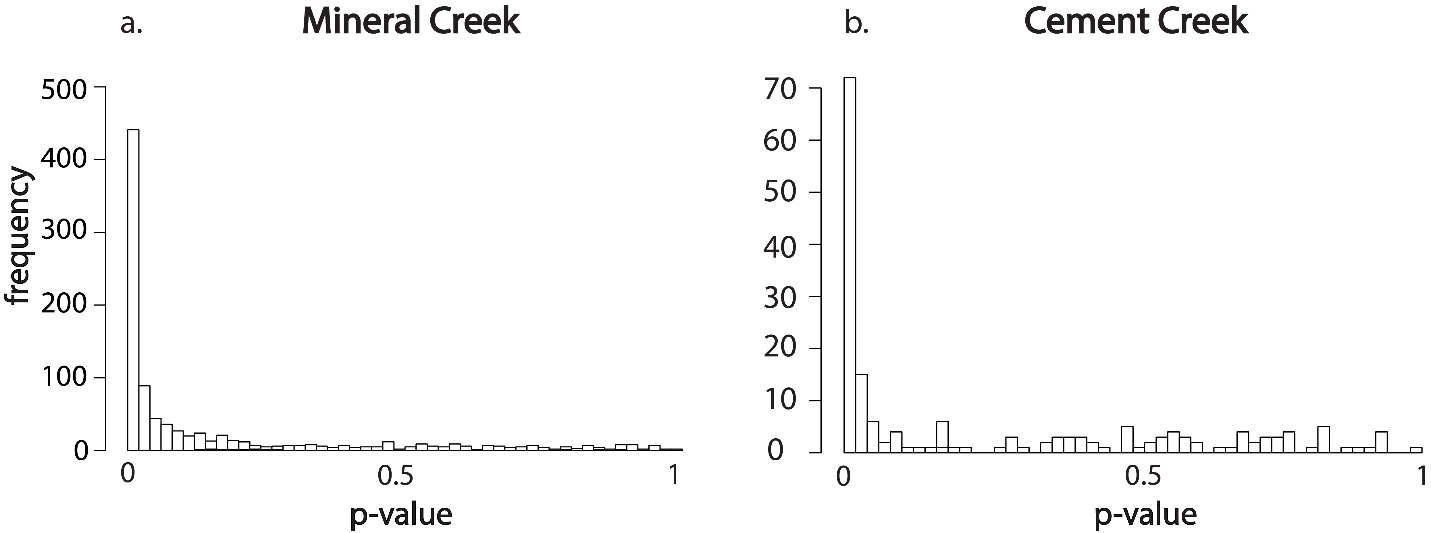


**Figure S10.** Corrected p-value distribution for the differential abundance analysis.


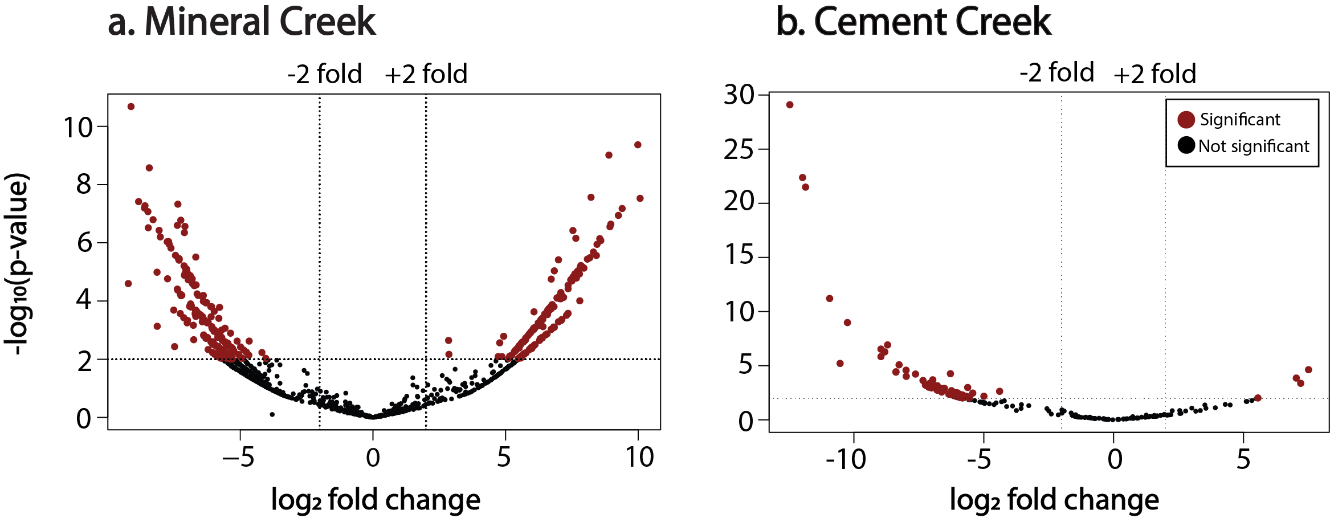


**Figure S11.** Volcano plots highlight differentially abundant taxa in porewaters and microcosm sediments for (a) Mineral Creek and (b) Cement Creek. The horizontal dotted line in (a) and (b) represents the p-value cutoff for significant taxa (*p* < 0.01) and the vertical dotted lines in (a) and (b) represent the log_2_fold change cutoff for significant taxa (2^-2^ and 2^2^ = ± 0.25). All taxa that exceeded these thresholds are considered significantly abundant and not due to random chance (i.e. all red points). Histograms of p-values associated with these plots are included in Figure S8.

**Figure S12.** Differentially abundant bacteria in microcosm sediments (positive log2fold change) and porewaters (negative log2fold change) for Mineral Creek classified by class and genus.

**Figure S13.** Differentially abundant bacteria in microcosm sediments (positive log2fold change) and porewaters (negative log2fold change) for Cement Creek classified by class and genus. No differentially abundant genes were classified at the species level.


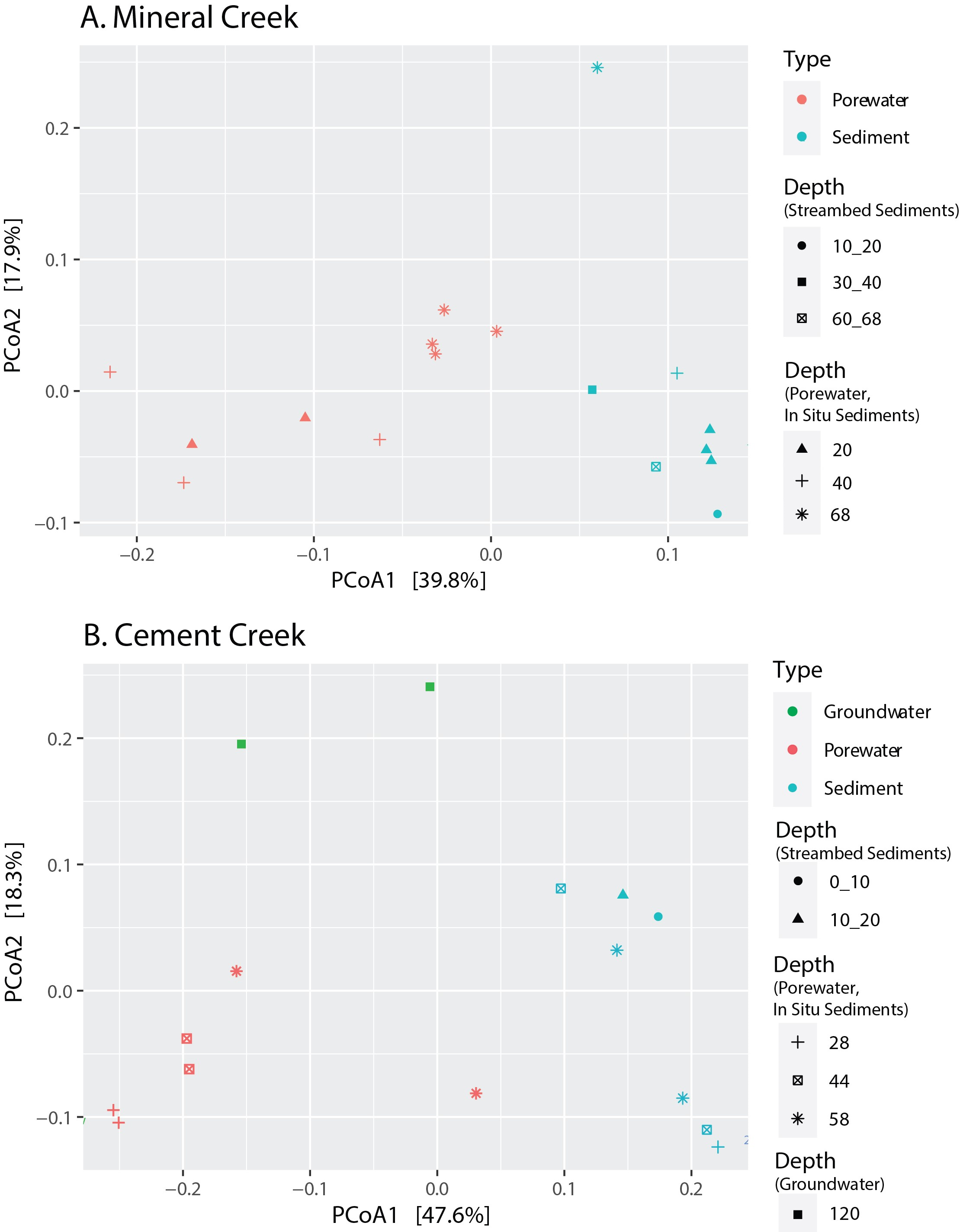


**Figure S14.** Beta diversity of streambed samples, in situ microcosm sediment samples collected after incubation in the well clusters, porewater samples, and groundwater samples.
